# Bimodal specificity of TF–DNA recognition in embryonic stem cells

**DOI:** 10.1093/nar/gkaf333

**Published:** 2025-04-26

**Authors:** Michael Povolotskii, Maor Yehezkehely, Oren Ram, David B Lukatsky

**Affiliations:** Department of Chemistry, Ben-Gurion University of the Negev, Beer-Sheva, 8410501, Israel; Department of Chemistry, Ben-Gurion University of the Negev, Beer-Sheva, 8410501, Israel; Department of Biological Chemistry, The Institute of Life Sciences, The Hebrew University of Jerusalem, Jerusalem, 91904, Israel; Department of Chemistry, Ben-Gurion University of the Negev, Beer-Sheva, 8410501, Israel

## Abstract

Transcription factors (TFs) bind genomic DNA regulating gene expression and developmental programs in embryonic stem cells (ESCs). Even though comprehensive genome-wide molecular maps for TF–DNA binding are experimentally available for key pluripotency-associated TFs, the understanding of molecular design principles responsible for TF–DNA recognition remains incomplete. Here, we show that binding preferences of key pluripotency TFs, such as Pou5f1 (Oct4), Smad1, Otx2, Srf, and Nanog, exhibit bimodality in the local GC-content distribution. Sequence-dependent binding specificity of these TFs is distributed across three major contributions. First, local GC-content is dominant in high-GC-content regions. Second, recognition of specific *k*-mers is predominant in low-GC-content regions. Third, short tandem repeats (STRs) are highly predictive in both low- and high-GC-content regions. In sharp contrast, the binding preferences of c-Myc are exclusively dominated by local GC-content and STRs in high-GC-content genomic regions. We demonstrate that the transition in the TF–DNA binding landscape upon ESC differentiation is regulated by the concentration of c-Myc, which forms a bivalent c-Myc-Max heterotetramer upon promoter binding, competing with key pluripotency factors such as Smad1. Finally, a direct interaction between c-Myc and key pluripotency factors is not required to achieve this transition.

## Graphical Abstract

**Graphical Abstract:**
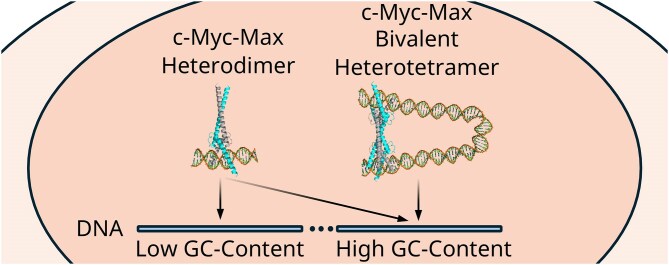

## Introduction

Transcription factors (TFs) bind genomic DNA regulating gene expression [1–7]. Using *Escherichia coli lac* repressor and λ phage repressor as model systems, seminal studies by Jacob-Monod [2] and Ptashne [7] established that short regulatory DNA sequences specifically bind TFs with high affinity. The subsequent studies by Riggs [4] and von Hippel [5] revealed that in the case of *lac* repressor, 98% or more of repressor is bound to DNA predominantly at sites other than the *lac* operator. It is explained by the fact that inducers shift repressor from operator to nonoperator DNA for significant distances but do not free it from DNA.

Currently, five decades after those studies, high-throughput methods, such as chromatin immunoprecipitation followed by massively parallel sequencing (ChIP-seq) allow genome-wide identification of TF–DNA binding maps [8–13]. Similar to how *lac* repressor binds nonoperator DNA, these maps reveal that the majority of variable TF–DNA binding events occur outside of known specific TF binding motifs [8, 9].

Even though molecular mechanisms responsible for specific TF–DNA recognition have been studied over the past 60 years, many questions still remain unanswered [14–18]. Understanding such mechanisms in eukaryotic cells is further complicated by the effect of chromatin structure, promoter–enhancer interactions, protein–protein interactions, and epigenetic modifications of histones and DNA [9, 19, 20].

Interestingly, genome-wide association studies (GWAS) show that the vast majority of disease-associated genetic variants are located in noncoding regions [19]. Understanding of molecular mechanisms responsible for TF recognition of such noncoding variable genomic regions constitutes one of the key open questions impeding the understanding of phenotypic consequences of disease-associated variants [19].

It is of particular importance to establish the link between TF–DNA recognition and gene expression programs in embryonic stem cells (ESCs) due to the similarities between ESCs with various cancers [12]. Specific TF–DNA binding motifs provide only a limited understanding of the experimentally determined TF–DNA binding network in ESCs [8]. Mechanistic understanding of this binding network is further complicated by the fact that different pluripotency TFs interact with each other forming multimeric protein complexes [12]. One such TF, c-Myc, is an important transcriptional regulator in ESCs, somatic cell reprogramming, and various cancers [12]. In particular, increased c-Myc expression level constitutes a common feature of undifferentiated ESCs and cancer cells [12]. In the past, it was revealed that the c-Myc-centered regulatory module is largely uncoupled, both in terms of protein–protein and TF–DNA binding, from the module comprising other key pluripotency factors, such as Nanog, Pou5f1 (Oct4), Sox2, and Smad1 [12]. In particular, it was shown that these two modules bind different genomic regions [12]. Molecular recognition mechanisms responsible for such behavior remain unknown.

Here, using human ESCs as a model system, we show that key pluripotency TFs possess bimodal intrinsic DNA recognition specificity characterized by fundamentally different DNA–binding mechanisms in low-GC-content (GC-poor) and high-GC-content (GC-rich) genomic regions, respectively. In particular, in GC-poor genomic regions, TF–DNA binding recognition is predominantly determined by specifically recognized *k*-mers. In contrast, in GC-rich genomic regions, TFs recognize the local genomic GC-content but possess a weak *k*-mer specificity. Nonconsensus repetitive elements contribute to TF-DNA recognition in both low- and high-GC-content regions.

We also demonstrate that upon developmental transitions, c-Myc can influence the TF–DNA recognition mode of key pluripotency factors, such as Smad1, Pou5f1 (Oct4), Otx2, and Srf. We provide further evidence that c-Myc competes with these pluripotency factors in GC-rich genomic regions, such as the vicinity of transcription start sites (TSSs). Using a simple chemical kinetics model, we predict that no direct interaction between c-Myc and the module comprising key pluripotency factors, such as Smad1, is required in order to achieve the transition in the TF–DNA binding landscape upon ESC differentiation. Rather this transition is regulated by the concentration of c-Myc, competing with other key pluripotency factors for promoter binding. We predict that the formation of a bivalent c-Myc-Max heterotetramer, for example, by looping DNA, is necessary for achieving this transition.

## Materials and methods

### MNChIP-seq data acquisition

We used micrococcal nuclease (MNase) based chromatin immunoprecipitation sequencing (MNChIP-seq) data obtained in undifferentiated human ESCs and during early stages of ESC differentiation: mesendoderm (dMS), endoderm (dEN), mesoderm (dME), and ectoderm (dEC) [8]. In our analyses, we used the processed BED-format data, which are publicly available at the Gene Expression Omnibus under the accession number GSE61475 [8]. In case of multiple replicas available for a given TF and a given developmental stage, we used the replica with the largest peak count. For the genomic analysis, we used the human reference genome version hg19. All accession numbers and peak counts of the chosen replicas are listed in Supplementary Table S1.

### Analysis of CpG islands

We used the coordinates of CpG islands in the hg19 human genome version (Supplementary Table S2). The data are publicly available at the Genome Browser (https://genome.ucsc.edu/cgi-bin/hgTables?db=hg19&hgta_group=regulation&hgta_track=cpgIslandExt&hgta_table=cpgIslandExt&hgta_doSchema=describe+table+schema).

### ATAC-seq data acquisition

To select control sequences for *k*-mer specificity evaluation in ESCs, we used transposase-accessible chromatin using sequencing (ATAC-seq) data [21]. The data are available at the Gene Expression Omnibus under the accession number GSM3163874 [21].

### Analysis of DNA methylation

To study the effect of DNA methylation on TF occupancy, we employed DNA methylation data from [22, 23], under the accession number GSE46644; we used the replicas GSM1112840_BiSeq_cpgMethylation_BioSam_1122_HUES64 from the GSE46644_bedFiles_set1 archive and GSM916051_BiSeq_cpgMethylation_HUES64_derived_ CD184_BioSam_705 from the GSE46644_bedFiles_set2 archive for ESCs and dEN, respectively.

### Gene expression data acquisition

We used the gene expression data from Supplementary Table S2 of [23].

### AlphaFold 3 modeling of c-Myc-Max-DNA complex

We used AlphaFold 3 [24] for predicting c-Myc-Max-DNA complex structures for a set of genomic DNA sequences (Supplementary Table S3). In simulations, we used truncated protein sequences of c-Myc and Max, representing the DNA-binding domain (DBD), analogous to the sequences used in experimentally obtained c-Myc-Max-DNA crystallographic structures [25] (Supplementary Table S3).

## Results

In what follows, we dissect sequence-dependent TF–DNA binding specificity into three major contributions: local GC-content, specific *k*-mers, and short tandem repeats (STRs). We refer to local GC-content and STRs as nonconsensus TF–DNA binding, and to specific *k*-mers as consensus TF–DNA binding. Intuitively, nonconsensus TF–DNA binding implies that different TFs, despite the fact that they possess entirely different specific, consensus TF–DNA binding motifs, can compete for commonly recognized DNA regions possessing certain nonconsensus elements. We now describe these contributions in detail.

### Dissecting TF-DNA binding specificity in relation to GC-content bimodality

**Figure 1.**
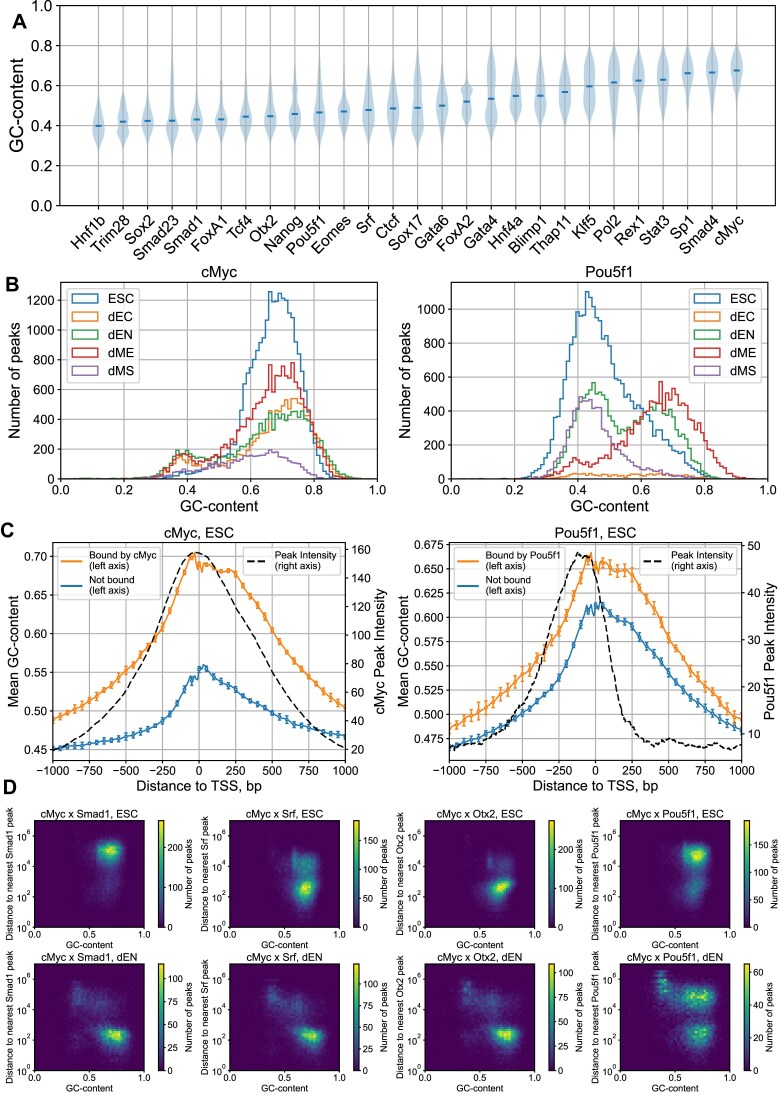
Dissecting TF–DNA binding specificity in relation to GC-content bimodality. (**A**) Violin plot of the GC-content distribution of MNChIP-seq peaks for 27 TFs in ESCs (data are taken from [8]). TFs are sorted by the median GC-content of peaks. (**B**) Example: Histogram of the GC-content distribution of MNChIP-seq peaks for c-Myc and Pou5f1 (Oct4) in different developmental stages. (**C**) Correlation between the mean local GC-content and the mean binding intensity of aligned MNChIP-seq peaks for c-Myc and Pou5f1 in ESCs as a function of genomic position relative to TSS. Orange curves show the mean GC-content for TSSs bound by c-Myc (left) and Pou5f1 (right). Blue curves show the mean GC-content for TSSs not bound by c-Myc (left) and Pou5f1 (right). We defined a given TSS as “bound” if it contains at least one binding peak within 1000-bp around the TSS. The local GC-content was computed within a sliding window with a width of 50-bp. For cMyc, the GC-content of bound TSSs shows a strong correlation with the average binding peak intensity, with the Pearson linear correlation coefficient $R = 0.97\ ( {p = 1.8 \times {{{10}}^{ - 118}}} )$. For Pou5f1, the correlation is weaker, with $R = 0.62\ ( {p = 1.9 \times {{{10}}^{ - 22}}} )$. Error bars represent one standard deviation of the mean GC-content, determined by dividing the entire set of TSSs into 10 equally sized subgroups and calculating the average GC-content for each subgroup. (**D**) The joint distribution of the GC-content in c-Myc binding peaks and their proximity to the nearest peaks of Smad1, Srf, Otx2, and Pou5f1, respectively, shown for two cell types, ESC and dEN. The distances are measured between peak centers. We partitioned the GC-content range (0 to 1) into 50 equal bins and the distance range (1 to 10^7^-bp) into 49 logarithmically scaled bins, counting the frequency of binding peaks within the bins.

Statistical analysis of the local GC-content of MNChIP-seq peaks shows a large degree of variability within and between different TFs (Fig. 1A). Key pluripotency factors, such as Nanog, Pou5f1 (Oct4), Sox2, Otx2, Smad1, and Srf are characterized by a median GC-content <0.5 in ESCs (Fig. 1A). Remarkably, a significant number of TFs show bimodality in the GC-content distribution (Fig. 1B and Supplementary Fig. S1). For example, Pou5f1, Smad1, Otx2, and Srf exhibit a transition from lower to higher GC-content upon developmental transition from ESCs to dEN (Fig. 1B and Supplementary Fig. S1). Other TFs, such as Nanog, exhibit a static bimodality in the GC-content distribution of MNChIP-seq peaks at different stages of differentiation (Supplementary Fig. S1). On the contrary, c-Myc is characterized by a stable local GC-content distribution with the highest median GC-content (Fig. 1A and B). Statistically, genomic regions around TSSs represent high-GC-content regions, as well as regions with high binding-peak intensity (Fig. 1C). For example, the mean peak intensity of c-Myc and Pou5f1 binding peaks aligned around TSSs correlates with the mean local GC-content (Fig. 1C).

The analysis of co-binding between c-Myc and other key pluripotency factors such as Smad1, Srf, Otx2, and Pou5f1 shows that in the course of differentiation, there is a competition between c-Myc and these TFs for binding GC-rich DNA regions (Fig. 1D and Supplementary Fig. S2). In particular, upon differentiation from ESCs to dEN, peaks of these TFs undergo a transition toward high-GC-content genomic regions, which were previously occupied by c-Myc (Fig. 1D and Supplementary Fig. S2). In what follows we show that this transition does not require a direct interaction of c-Myc with these TFs.

### Key pluripotency TFs bind genomic regions depleted of DNA methylation

Considering the fact that GC-rich genomic regions bound by key pluripotency TFs near TSSs are statistically enriched in CpG islands (Supplementary Fig. S3), we aim to test the propensity of these TFs for binding methylated CpG sites. To this end, using methylation data from [22, 23], we calculated the average DNAme level within 4-kb-wide genomic regions aligned by the center of MNChIP-seq binding peaks of the selected TFs (c-Myc, Nanog, Otx2, Pou5f1, Smad1, Sox2, Srf, and Tcf4) in two cell types, ESCs and dEN (Supplementary Fig. S4). The methylation level was normalized by the total number of CpG pairs located at a given position relative to the peak center. The results demonstrate a decrease in average DNAme level within the peaks of all eight TFs that we analyzed, compared to genomic regions outside the peaks. This shows that key pluripotency TFs, including c-Myc and Smad1, possess a preference for unmethylated DNA in both undifferentiated ESCs and in dEN (Supplementary Fig. S4).

### Bimodality of *k*-mer specificity is linked to bimodality of GC-content

The second feature responsible for TF-DNA binding specificity we consider (in addition to the local GC-content) is short genomic sequences of length *k*. We term such sequences *k*-mers. To assess TF specificity to *k*-mers, we developed a peak-over-background statistical energy model, according to which, the binding energy (in the units of *k*_B_*T*, where *k*_B_ is the Boltzmann constant and *T* is the temperature) of a particular *k*-mer named *s* is calculated as follows:


(1) \begin{eqnarray*}
\begin{array}{*{20}{c}} {U_k^{\left( s \right)} = - \ln \frac{{{{{\left\langle {N_k^{\left( s \right)}} \right\rangle }}_{{\rm peak}}} + 1}}{{{{{\left\langle {N_k^{\left( s \right)}} \right\rangle }}_{{\rm background}}} + 1}}} \end{array}
\end{eqnarray*}

Here, ${{\langle {N_k^{\ ( s )}} \rangle }_{{\rm peak}}}$ denotes how many times, on average, the *k*-mer $s$ and its reverse complement occur in the 100 bp-long nucleotide sequences at the center of MNChIP-seq binding peaks. The denominator, ${{\langle {N_k^{\ ( s )}} \rangle }_{{\rm background}}}$, is computed for control sequences, which come from 100-bp-long genomic regions situated 100-bp from the binding peak edges, and not overlapping with other peaks. Adding 1 to both nominator and denominator avoids taking the logarithm of zero and dividing by zero.

**Figure 2.**
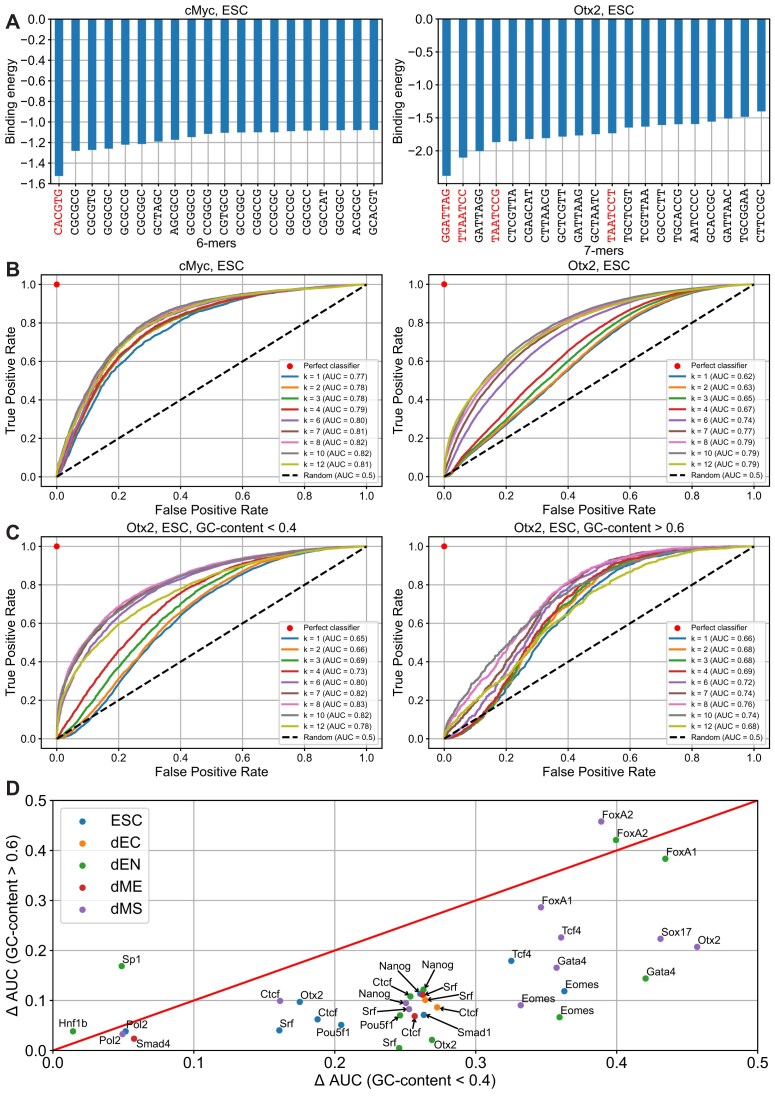
Characterizing *k*-mer specificity of TFs. (**A**) Examples of *k*-mers sorted by increasing binding energy (in the units of *k*_B_*T*) within the proposed peak-over-background energy model for c-Myc (*k*= 6) and Otx2 (*k*= 7). According to the model, the binding energy of each *k*-mer is defined by Eq. (1). MNChIP-seq data derived from [8], we used 80% of it as a “training subset” to assess binding energies of *k*-mers. The c-Myc binding consensus binding motif, *CACGTG*, is highlighted in red on the left plot, and the 7-mers containing the Otx2 consensus binding motif, *GGATTA*, or its reverse complement, *TAATCC*, are highlighted in red on the right plot. (**B**) ROC curves visualize the ability of the proposed energy model to predict c-Myc and Otx2 binding peaks depending on *k*-mer length. The prediction of whether the sequence belongs to the peak or the background is based on the value of its free energy defined by Eq. (2). We precomputed binding energies using the training dataset consisting of 80% of the entire dataset, as described in panel (A). To evaluate the model performance, we compiled the testing set, using the remaining 20% of the entire dataset. The *x*-axis shows a FPR, and the *y*-axis shows a TPR. Pairs of TPR and FPR at different thresholds constitute a ROC curve for each *k*-mer length. The diagonal dotted line signifies classifiers randomly assigning peak status at a consistent probability, equating TPR with FPR. A perfect classifier is represented by a red dot at the coordinates (0,1). The area under the ROC curve (AUC) provides a probabilistic estimate for a randomly selected peak to have lower free energy than a randomly selected background sequence. Higher AUC values indicate superior predictive capability. (**C**) Same as panel (B), but instead of analyzing MNChIP-seq peaks of two different TFs, we picked out two groups of Otx2 peaks, the first group consisting of peaks with GC-content <0.4 and the second one consisting of peaks with GC-content >0.6. All the procedures including splitting into training and testing subsets, *k*-mer binding energy assessment, free energy calculation, and ROC curves visualization were performed separately for each group. (**D**) Summary of *k*-mer binding specificity depending on the GC-content. Using the same procedure as in panel (C) for each TF and each cell type, we compiled two groups of MNChIP-seq peaks with GC-content below 0.4 and above 0.6, respectively. Next, we calculated the area under the ROC curve (AUC) in both groups for different *k*-mer length, *k*, and then computed *k*-mer specificity characteristic, ΔAUC, using Eq. (3). The pairs of ΔAUC values for different GC-content were used as coordinates to build a scatter plot. Each point is labeled by the corresponding TF, and the developmental stage is encoded by color. If the number of peaks in either of the two groups is <1000, the point is not shown. The overwhelming majority of proteins, 32 out of 36, fall below the diagonal.

For a given TF in a given developmental stage, we used 80% of all MNChIP-seq peaks as a training subset to assess ${{\langle {N_k^{\ ( s )}} \rangle }_{{\rm peak}}}$ and ${{\langle {N_k^{\ ( s )}} \rangle }_{{\rm background}}}$. The examples of *k*-mers with the least binding energy calculated using Eq. (1) for two TFs, c-Myc and Otx2, are shown in Fig. 2A. As expected, their known binding motifs [8] have the lowest binding energy (Fig. 2A).

The remaining 20% of MNChIP-seq peaks were used as a testing subset to evaluate the model performance. To do so, for each *k*-mer length, *k*, we predicted whether a given sequence from the testing subset belongs to the peak or the background based on the value of its free energy (in the units of *k*_B_*T*) defined as:


(2) \begin{eqnarray*}
\begin{array}{*{20}{c}} {{{F}_k} = - ln\mathop \sum \limits_{i = 1}^{L - k + 1} {\mathrm{exp}}\left( { - U_k^{\left( {{{s}_i}} \right)}} \right)} \end{array}
\end{eqnarray*}

Here, *L* is the length of each sequence and is equal to 100-bp. $U_k^{( {{{s}_i}} )}$ is the precomputed binding energy of a given *k*-mer, ${{s}_i}$, made up of the sequence nucleotides with indices from *i* to *i*+ *k*-1. The lower the free energy of a sequence is, the more likely the sequence belongs to a binding peak. Notably, in the case of *k*= 1, binding energies are assigned to single nucleotides, meaning that the free energy depends on the local GC-content only.

In order to evaluate the predictive ability of the free energy model, Eq. (2), we employ area under the curve of the receiver operating characteristic (ROC AUC) [26]. Specifically, for any free energy threshold ${{F}_t}$ which separates peaks from the background, there is a true positive rate (TPR) which is the fraction of peak sequences having the free energy below this threshold and thus correctly classified as peaks (true positives), and a false positive rate (FPR) which is the proportion of background sequences having the free energy below the threshold and thus misidentified as peaks (false positives). Pairs of FPR and TPR at different thresholds constitute a ROC curve for each length of *k*-mers. The area under the ROC curve (AUC) provides a probabilistic estimate of a randomly selected peak sequence having lower free energy than a randomly selected background sequence. Higher values of AUC indicate a superior predictive capability.

We compared how ROC AUC depends on the *k*-mer length in the case of different TFs (Fig. 2B). For TFs with high average GC-content of their binding peaks, such as c-Myc (Fig. 1B), there was only a slight increase in ROC AUC computed for *k* > 1 compared to *k* = 1. Therefore, c-Myc represents the case where the local GC-content provides a dominant contribution to the TF–DNA binding free energy. In contrast, TFs with low average GC-content of their binding peaks, such as Otx2 in ESCs, are characterized by a much larger difference of ROC AUC for *k* > 1 compared to *k* = 1 (Fig. 2B).

In the next step, we dissected all Otx2 peaks into two groups. These groups correspond to binding peaks of Otx2 in ESCs with GC-content <0.4 and >0.6, respectively. Next, we used these groups as two separate datasets, and evaluated ROC AUC using these datasets (Fig. 2C). Here we observed that GC-poor peaks of Otx2 are characterized by a larger difference of ROC AUC between *k* > 1 and *k* = 1 (thus being more *k*-mer-specific), as compared to the case of GC-rich peaks characterized by a smaller difference of ROC AUC between *k* > 1 and *k* = 1 (Fig. 2C).

To assess the generality of this phenomenon, we repeated the same procedure with different TFs in different cell types, provided there were at least 1000 peaks in both GC-poor and GC-rich groups. To characterize the TF *k*-mer specificity with a single value, we introduced the ΔAUC value, defined as the difference between maximal AUC among all *k*-mer lengths and AUC for *k*= 1:


(3) \begin{eqnarray*}
\begin{array}{*{20}{c}} {\Delta {\mathrm{AUC}} = \mathop {\max }\limits_k {\mathrm{AUC}}\left( k \right) - {\mathrm{AUC}}\left( 1 \right)} \end{array}
\end{eqnarray*}

The subtraction of ${\mathrm{AUC}}( 1 )$ serves the purpose of the GC-content normalization. For each TF and each developmental stage, we calculated $\Delta {\mathrm{AUC}}$ using subsets with GC-content <0.4 and >0.6, respectively (Fig. 2D). We reveal here that the majority of TFs are characterized by larger $\Delta {\mathrm{AUC}}$ for GC-poor peaks as compared to GC-rich peaks, suggesting the generality of the discovered phenomenon. Notably, Sp1 and FoxA2 represent two outliers showing slightly higher *k*-mer specificity in GC-rich regions as compared to GC-poor regions (Fig. 2D). We emphasize that c-Myc is not represented in Fig. 2D due to the fact that the GC-content distribution of c-Myc is shifted toward high GC-content values in all developmental stages (i.e. very few peaks with low GC-content, Fig. 1B), and c-Myc is thus characterized by a low *k*-mer specificity (Fig. 2B).

In order to further validate the generality of our results, we repeated $\Delta {\mathrm{AUC}}$ calculations using an alternative selection of background regions. In particular, we used the available ATAC-seq data [21] in ESCs to select alternative background regions (Supplementary Fig. S5). Among all available ATAC-seq peaks, we selected only peaks with GC-content <0.4 and >0.6, in order to match the GC-content of the corresponding MNChIP-seq subsets. We discarded ATAC-seq peaks which overlapped with MNChIP-seq peaks. As a result of this procedure, we obtained that out of 9 TFs, only Pol2 has a larger $\Delta {\mathrm{AUC}}$ for GC-rich peaks compared to GC-poor peaks (Supplementary Fig. S5). This further supports the conclusion that GC-rich genomic regions bound by TFs are characterized by a lower *k*-mer specificity compared to GC-poor regions.

Lower *k*-mer specificity of GC-rich genomic regions compared to GC-poor regions, implies a stronger contribution of nonconsensus TF–DNA binding to TF–DNA recognition specificity in these GC-rich regions. By using the term “nonconsensus” binding we mean the contribution of the local GC-content (described above) and nonconsensus STRs described in the next section. Intuitively, nonconsensus TF–DNA binding implies that different TFs, despite the fact that they possess entirely different specific, consensus TF–DNA binding motifs, can compete for commonly recognized DNA regions possessing certain nonconsensus elements.

We emphasize that nonconsensus binding does not necessarily imply the absence of established binding motifs. Rather, the main characteristic of nonconsensus TF–DNA binding described in this section is a larger contribution of the local GC-content to binding specificity (in GC-rich regions) compared to *k*-mers, which include consensus binding motifs but are not limited to them. This is illustrated by the analysis of consensus motifs inside MNChIP-seq peaks of c-Myc, Smad1, and Otx2 (Supplementary Fig. S6). In particular, in GC-rich regions ∼14% of c-Myc peaks, ∼59% of Smad1 peaks, and ∼20% of Otx2 peaks contain at least one specific, consensus motif. However, as ROC curves demonstrate, the major contribution to TF–DNA binding specificity in GC-rich regions is provided by the local GC-content rather than consensus motifs (Supplementary Fig. S6).

### Effect of short tandem repeats on TF–DNA binding specificity

The third local genomic sequence feature that we analyze is STRs. In order to test the effect of STRs on TF–DNA binding specificity, we use pair correlation functions developed previously [27–29]. These correlation functions represent the probability of finding two nucleotides of a given type separated by relative distance *x*. In particular, for each DNA sequence in a sequence set we compute:


(4) \begin{eqnarray*}
\begin{array}{*{20}{c}} {{{\eta }_{\alpha \beta }}\left( x \right) = \frac{{{{N}_{\alpha \beta }}\left( x \right) - {{{\left\langle {{{N}_{\alpha \beta }}\left( x \right)} \right\rangle }}_{rand}}}}{L}} \end{array}
\end{eqnarray*}

Here, ${{N}_{\alpha \beta }}( x )$ is the total number of nucleotide pairs of the types *α* and *β*, separated by the relative distance *x*, ${{\langle {{{N}_{\alpha \beta }}( x )} \rangle }_{rand}}$ is the corresponding average number in the randomized sequence set, and $L$ is the length of the DNA sequence. The randomization procedure randomly reshuffles each DNA sequence, keeping the GC-content of each sequence intact. The averaging, ${{\langle {{{N}_{\alpha \beta }}( x )} \rangle }_{rand}}$, is performed over 100 random realizations of the original sequence. This randomization procedure normalizes the varying genomic GC-content, allowing us to compare symmetry properties of DNA sequences from different genomic locations characterized by a variable average GC-content. At the end of this procedure, for each value of the relative distance *x*, we average the computed ${{\eta }_{\alpha \beta }}( x )$ over all the DNA sequences in the set.

**Figure 3.**
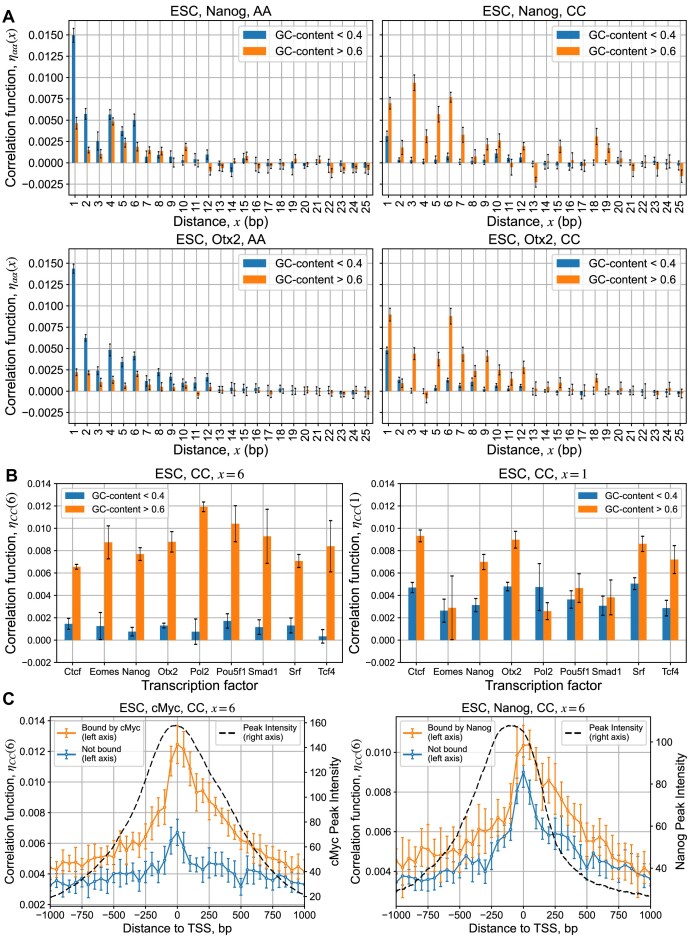
Analysis of genomic repetitive DNA sequence elements depending on the GC-content. (**A**) Correlation functions are computed according Eq. (4) for the two groups of genomic DNA sequences as a function of the relative distance *x* between the pair of nucleotides of a given type (A or C). The first group contains the 100-bp-long genomic sequences of undifferentiated ESCs extracted from the center of MNChIP-seq peaks for Nanog (top) and Otx2 (bottom) with GC-content <0.4, and the second group contains MNChIP-seq peaks with GC-content >0.6. To compute error bars for each sequence group, we divide the group into ten subgroups with an equal number of sequences in each subgroup and compute the mean correlation functions, *η*_AA_ (*x*) and *η*_CC_ (*x*), for each subgroup. Next, we compute the standard deviation of the mean correlation functions between subgroups. Error bars represent one standard deviation in each direction around the mean (i.e. two standard deviations overall). (**B**) Values of the correlation function *η*_CC_ (*x*) computed for the same groups as in (A) for various TFs at the selected distances, *x*= 6 and *x*= 1. The error bars are computed the same way as in (A). (**C**) The correlation between the mean value of the correlation function, *η*_CC_ (6), and the mean binding peak intensity for c-Myc and Nanog near TSSs. We categorized regions into those bound (orange line) or unbound (blue line) by the TF, defining “bound” as having at least one binding peak within 1000 bp of the TSS, measured from the peak’s nearest edge. The value of *η*_CC_ (6) was computed using a 50-bp-wide sliding window and then averaged for regions aligned by the TSS. Dashed line represents the corresponding mean peak intensity for bound TSS regions. Error bars represent one standard deviation of the mean *η*_CC_ (6), determined by dividing the genomic regions into 10 equally sized subgroups and calculating the average for each subgroup. For c-Myc, the Pearson linear correlation coefficients between the average peak intensity and *η*_CC_ (6): $R = 0.92\ ( {p = 9.1 \times {{{10}}^{ - 18}}} )$ and $R = 0.82\ ( {p = 6.3 \times {{{10}}^{ - 11}}} )$ for TSS regions bound and unbound by c-Myc, respectively. For Nanog, these values: $R = 0.71\ ( {p = 2.1 \times {{{10}}^{ - 7}}} )$ for TSS regions both bound and unbound by Nanog.

Here, we analyzed repeat symmetries inside 100-bp-long genomic regions in the center of MNChIP-seq binding peaks of different TFs in different developmental stages, dividing peaks into GC-poor and GC-rich groups, similar to what we performed for the *k*-mer specificity analysis (Fig. 3A and B). Notably, the correlation function ${{\eta }_{CC}}( x )$ at *x*= 6 (this corresponds to repetitive pattern [CNNNNNC]) provides the strongest distinction between GC-rich and GC-poor regions for a significant number of TFs (Fig. 3B). For GC-poor regions, the correlation function ${{\eta }_{AA}}( x )$ is strongly increased at *x*= 1 (i.e. enriched in poly(A) tracks) (Fig. 3A). In addition, we aligned 2000 bp-long genomic regions near TSSs dividing them into two groups, bound and unbound by the TF, similar to what we performed for the GC-content specificity analysis, and calculated pair correlation functions using 50-bp sliding window (Fig. 3C). For the two shown examples of c-Myc and Nanog, ${{\eta }_{CC}}( 6 )$ shows an excellent correlation with the mean binding intensity of bound peaks (Fig. 3C).

Overall, our results demonstrate that for a significant number of TFs, different nonconsensus repetitive DNA sequence elements contribute to TF–DNA binding specificity in GC-rich [the strongest repetitive pattern, [CNNNNNC]/[GNNNNNG]], and GC-poor [the strongest repetitive pattern, poly(A)/poly(T)] genomic regions, respectively.

### c-Myc binds GC-rich genomic regions strongly depleted of antinucleosomal DNA sequences

**Figure 4.**
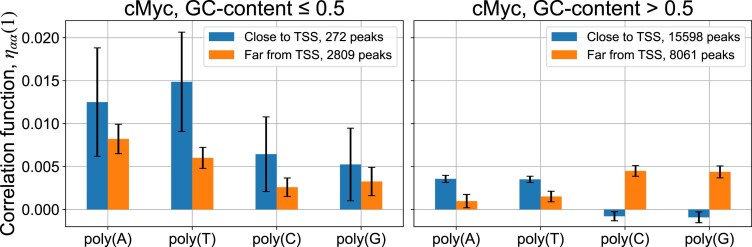
Antinucleosomal DNA sequences are strongly depleted in GC-rich genomic regions bound by c-Myc, compared with GC-poor regions bound by c-Myc and enriched in antinucleosomal sequences. Computed *η*_AA_ (1), *η*_TT_ (1), *η*_CC_ (1), and *η*_GG_ (1) [representing the enrichment of poly(A), poly(T), poly(C), and poly(G)] for c-Myc MNChIP-seq peaks compiled from all five developmental stages of ESCs. Correlation functions were computed for 100-bp-long DNA sequences extracted from the center of peaks. In case of overlapping peaks from different stages, we included only a peak with the leftmost start coordinate. The peaks shorter than 100-bp were omitted. Left and right plots represent correlation functions computed for GC-poor (GC-content < 0.5) and GC-rich (GC-content > 0.5) c-Myc MNChIP-seq peaks, respectively. Peaks located within the interval ± 1000-bp are termed “close to TSS.” Peaks located outside of the interval ± 1000-bp around TSS are termed “far from TSS.” To compute error bars for each sequence group, we randomly split the group into ten subgroups with an equal number of sequences in each subgroup and computed the mean correlation functions, *η*_αα_ (1), for each subgroup. Error bars represent one standard deviation in each direction around the mean.

It was established in the past that poly(A)/poly(T) and poly(C)/poly(G) tracts represent antinucleosomal DNA sequences characterized by a high intrinsic rigidity [30–32]. Using Eq. (4), we analyzed the enrichment of poly(A), poly(T), poly(C), and poly(G) tracts in c-Myc binding regions by calculating pair correlation functions, ${{\eta }_{{\mathrm{AA}}}}( 1 )$, ${{\eta }_{{\mathrm{TT}}}}( 1 )$, ${{\eta }_{{\mathrm{CC}}}}( 1 )$, and ${{\eta }_{{\mathrm{GG}}}}( 1 )$, as described in the previous section (Fig. 4). The fact that these correlation functions are normalized by the GC-content [Eq. (4)] allows us to compare the relative enrichment of poly(A), poly(T), poly(C), and poly(G) tracts in MNChIP-seq peaks with a variable GC-content (Fig. 4).

**Figure 5.**
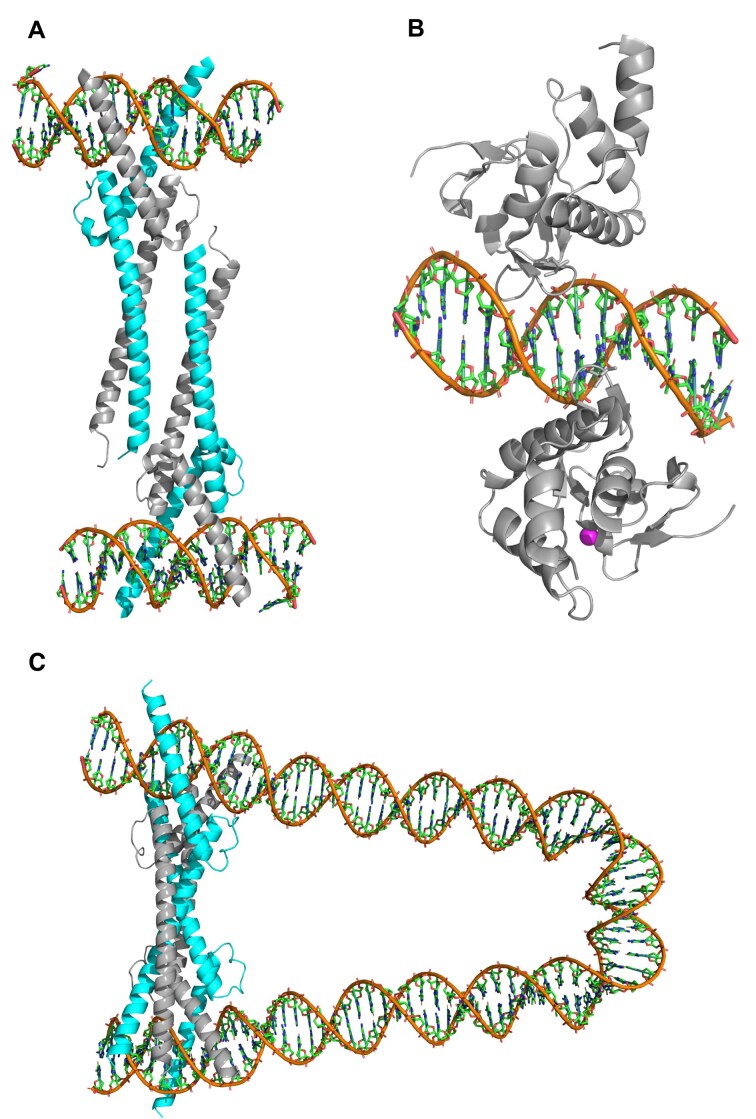
Crystallographic structures of truncated c-Myc-Max bivalent heterotetramer (**A**) and truncated Smad1 homodimer (**B**). The cyan and gray colors in panel (A) represent Myc and Max, respectively. Zink ion is shown in magenta in panel (B). These structures correspond to Protein Data Bank (PDB) accession numbers 1NKP (25) and 3KMP (34), respectively. (**C**) AlphaFold3 prediction of the c-Myc-Max bivalent heterotetramer bound to DNA (sequence ID 5186, Supplementary Table S3), with the length of 96-bp and the GC-content of 54.17%.

Here, we notice the strong deficiency of antinucleosomal poly(C)/poly(G) tracts in GC-rich genomic regions bound by c-Myc (∼89% of all c-Myc peaks), indicating a lower rigidity of these sequences (Fig. 4, right). In sharp contrast, GC-poor regions bound by c-Myc (∼11% of all c-Myc peaks) are enriched in antinucleosomal poly(A)/poly(T) tracts, indicating a higher rigidity of these sequences (Fig. 4, left). This strongly supports our working hypothesis (formulated in the next section) that c-Myc forms a bivalent c-Myc-Max heterotetramer in GC-rich genomic regions by looping DNA (Fig. 5), while GC-poor DNA sequences are bound by c-Myc forming a canonical c-Myc-Max heterodimer.

The observed depletion of poly(C) and poly(G) tracts in GC-rich DNA regions bound by c-Myc is particularly pronounced in the vicinity of TSS, where the majority (∼66%) of GC-rich c-Myc peaks are located (Fig. 4). Such a property represents a general feature of GC-rich regions bound by other TFs, with c-Myc exhibiting the strongest depletion (Supplementary Fig. S7).

### Model for competitive Myc-DNA binding with formation of bivalent heterotetramer

**Figure 6.**
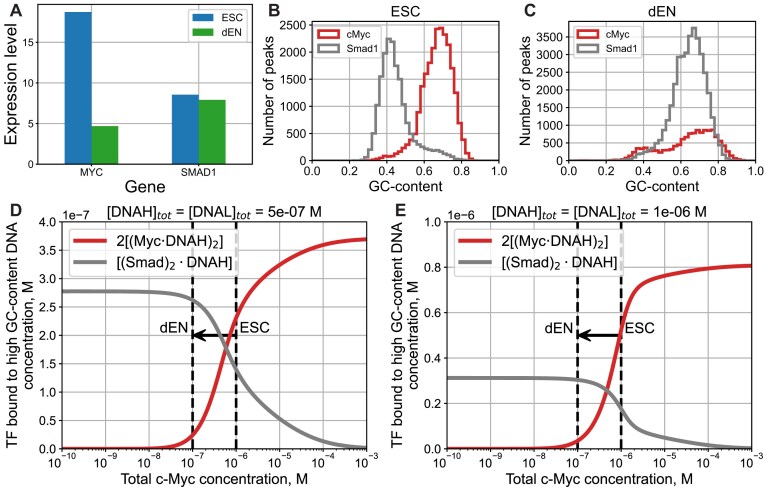
Model for competitive binding of c-Myc and Smad1 to high-GC DNA sequences. (**A**) Comparison of the c-Myc and Smad1 gene expression level in ESCs (blue) and dEN (green). The gene expression data are adopted from Supplementary Table S2 of [23]. (**B** and **C**) Comparison of the GC-content distribution across binding peaks of Myc (red) and Smad1 (gray) in ESCs (B) and dEN (C). We binned the GC-content range (0 to 1) into 100 equal segments, tallying the occurrence of binding peaks within these intervals. (**D** and **E**) The computed concentration of the high-GC DNA (DNAH) bound by bivalent Myc-Max heterotetramer and by Smad1 homodimer are shown as a function of the total Myc concentration, using two values of the total DNAH and DNAL concentrations, [DNAH]*_tot_*=[DNAL]*_tot_*= 0.5 ${\mathrm{\mu M}}$ (D), and [DNAH]*_tot_*=[DNAL]*_tot_*= 1 ${\mathrm{\mu M}}$ (E). The following values were used for the remaining model parameters: ${{[ {{\mathrm{Smad}}} ]}_{tot}} = 1{\mathrm{\mu M}}$, ${{K}_1} = 145{\mathrm{nM}}$, ${{K}_2} = 90{\mathrm{nM}}$, ${{K}_L} = 500{\mathrm{nM}}$, ${{\widetilde{K}}_H} = {{10}^{ - 14}}\ {{{\mathrm{M}}}^2}$, ${{\widetilde{K}}_L} = 2.5 \cdot {{10}^{ - 14}}\ {{{\mathrm{M}}}^2}$. Dashed lines qualitatively represent c-Myc concentrations in ESCs (right) and dEN (left), respectively. The arrow illustrates the developmental transition from ESCs (with a higher c-Myc concentration) to dEN (with a lower c-Myc concentration).

Here, we present support for our key working hypothesis that the transitions in the GC-content specificity of key pluripotency factors upon developmental transitions can be explained assuming that they compete with c-Myc for binding GC-rich genomic regions, without the need for direct interactions of these TFs with c-Myc. Previously, it has been established that c-Myc binding to genomic DNA, and its function in transcriptional activation requires heterodimerization with Max [25, 33]. In a seminal study by Nair and Burley [25], it has been shown that upon DNA binding, c-Myc-Max heterodimer can form a bivalent heterotetramer (Fig. 5). AlphaFold3 modeling [24] further supports the formation of a bivalent c-Myc-Max heterotetramer (Fig. 5C and Supplementary Table S3). Using c-Myc and Smad1 as a model system, here we show that a simple model for competitive TF–DNA binding qualitatively accounts for the observed distribution of Smad1 between high-GC-content (we term these regions as DNAH) and low-GC-content (we term these regions as DNAL) genomic regions (Fig. 6). In particular, in ESCs, the GC-content distribution of Smad1 MNChIP-seq peaks is strongly shifted toward low-GC-content genomic regions (DNAL) (Fig. 6B). On the contrary, in dEN the GC-content distribution of Smad1 MNChIP-seq peaks is strongly shifted toward high-GC-content genomic regions (DNAH) (Fig. 6C). In sharp contrast with Smad1, the GC-content distribution of c-Myc peaks is shifted toward high-GC-content genomic regions (DNAH) in both ESCs and dEN (Fig. 6B and C). Importantly, while the expression level of c-Myc is four times higher in ESCs than in dEN, the Smad1 expression level is practically the same in ESCs and dEN (Fig. 6A and see Supplementary Table S2 in [23]). Here, we show that the competition between c-Myc bivalent heterotetramer and Smad1 for genomic binding (without assuming any direct interaction between c-Myc and Smad1) can explain the experimentally observed transition of Smad1 genomic preferences upon developmental transition from ESCs to dEN (Fig. 6B and C).

Consider the binding of c-Myc-Max heterodimer to low-GC (DNAL) and high-GC (DNAH) DNA sequences. In what follows, we denote c-Myc-Max heterodimer as Myc. The following reactions describe the binding of Myc to DNA:


(5) \begin{eqnarray*}
{\mathrm{Myc}} + {\mathrm{DNAH}} &\mathop{\longleftrightarrow} \limits^{{{K}_1}}& \left[ {{\mathrm{Myc}} \cdot {\mathrm{DNAH}}} \right],
\end{eqnarray*}

(6) \begin{eqnarray*}
\left[ {{\mathrm{Myc}} \cdot {\mathrm{DNAH}}} \right] + \left[ {{\mathrm{Myc}} \cdot {\mathrm{DNAH}}} \right] &\mathop{\longleftrightarrow} \limits^{{{K}_2}}& \left[ {{\mathrm{\ }}{{{\left( {{\mathrm{Myc}} \cdot {\mathrm{DNAH}}} \right)}}_2}} \right],\nonumber\\
\end{eqnarray*}

(7) \begin{eqnarray*}
{\mathrm{Myc}} + {\mathrm{DNAL}} &\mathop{\longleftrightarrow} \limits^{{{K}_L}}& \left[ {{\mathrm{Myc}} \cdot {\mathrm{DNAL}}} \right],
\end{eqnarray*}

where ${{K}_1}$, ${{K}_2}$, and ${{K}_L}$ are the equilibrium dissociation constants. Here, [DNAH] is the concentration of the free (i.e. unbound) high-GC DNA, [DNAL] is the concentration of the free low-GC DNA, [Myc⋅DNAH] and [Myc⋅DNAL] is the concentration of high-GC and low-GC DNA bound by c-Myc-Max heterodimer, respectively; [(Myc⋅DNAH)_2_] is the concentration of a bivalent c-Myc-Max heterotetramer (Fig. 5). We assume that bivalent c-Myc-Max heterotetramer can be formed only on DNAH but not on DNAL. In contrast, we assume that c-Myc-Max heterodimer can bind both DNAH and DNAL. This constitutes the key assumption of our model. This assumption will allow us, first, to explain the fact that the vast majority of c-Myc–DNA binding peaks are located in high-GC genomic regions (Fig. 1B). Second, it will allow us to reproduce a sharp transition in Smad1 binding preferences upon the developmental transition from ESCs to dEN (Fig. 6). Supporting this assumption, AlphaFold3 simulations performed for DNA sequences with a varying GC-content show a weak but statistically significant correlation between the GC-content and the fraction of genomic DNA sequences forming a bivalent c-Myc-Max heterotetramer through DNA looping (Supplementary Fig. S8). This assumption is additionally supported by our finding that GC-rich genomic regions bound by c-Myc are strongly depleted of antinucleosomal poly(C)/poly(G) tracts, thus favoring DNA looping in these GC-rich regions. On the contrary, GC-poor genomic regions bound by c-Myc are enriched in antinucleosomal poly(A)/poly(T) tracts, thus disfavoring DNA looping in these GC-poor regions (Fig. 4).

We now describe competitive Smad1 binding to DNAH and DNAL genomic regions, respectively:


(8) \begin{eqnarray*}
2{\mathrm{\ Smad}} + {\mathrm{DNAH}} &\mathop{\longleftrightarrow} \limits^{{{{\widetilde{K}}}_H}}& \left[ {{{{\left( {{\mathrm{Smad}}} \right)}}_2} \cdot {\mathrm{DNAH}}} \right],
\end{eqnarray*}

(9) \begin{eqnarray*}
2{\mathrm{\ Smad}} + {\mathrm{DNAL}} &\mathop{\longleftrightarrow} \limits^{{{{\widetilde{K}}}_L}}& \left[ {{{{\left( {{\mathrm{Smad}}} \right)}}_2} \cdot {\mathrm{DNAL}}} \right],
\end{eqnarray*}

where ${{\widetilde{K}}_H}$ and ${{\widetilde{K}}_L}$ are the equilibrium dissociation constants for Smad1 binding to DNAH and DNAL genomic regions, respectively, and we assume that Smad1 binds both DNAH and DNAL as a homodimer [34].

The corresponding rate equations for Myc:


(10) \begin{eqnarray*}
\frac{{d\left[ {{\mathrm{Myc}} \cdot {\mathrm{DNAH}}} \right]}}{{dt}} &=& {{k}_1}\left[ {{\mathrm{Myc}}} \right]\left[ {{\mathrm{DNAH}}} \right] \nonumber\\ &-& {{k}_{ - 1}} \left[ {{\mathrm{Myc}} \cdot {\mathrm{DNAH}}} \right],
\end{eqnarray*}

(11) \begin{eqnarray*}
\frac{{d\left[ {{{{\left( {{\mathrm{Myc}} \cdot {\mathrm{DNAH}}} \right)}}_2}} \right]}}{{dt}} &=& {{k}_2}{{\left[ {{\mathrm{Myc}} \cdot {\mathrm{DNAH}}} \right]}^2} \nonumber\\ &-& {{k}_{ - 2}} \left[ {{{{\left( {{\mathrm{Myc}} \cdot {\mathrm{DNAH}}} \right)}}_2}} \right],
\end{eqnarray*}

(12) \begin{eqnarray*}
\frac{{d\left[ {{\mathrm{Myc}} \cdot {\mathrm{DNAL}}} \right]}}{{dt}} &=& {{k}_L}\left[ {{\mathrm{Myc}}} \right]\left[ {{\mathrm{DNAL}}} \right] \nonumber\\ &-& {{k}_{ - L}} \left[ {{\mathrm{Myc}} \cdot {\mathrm{DNAL}}} \right],
\end{eqnarray*}

For Smad1 binding we obtain the following:


(13) \begin{eqnarray*}
\frac{{d\left[ {{{{\left( {{\mathrm{Smad}}} \right)}}_2} \cdot {\mathrm{DNAH}}} \right]}}{{dt}} &=& {{\widetilde{k}}_H}{{\left[ {{\mathrm{Smad}}} \right]}^2}\left[ {{\mathrm{DNAH}}} \right] \nonumber\\ &-& {{\widetilde{k}}_{ - H}} \left[ {{{{\left( {{\mathrm{Smad}}} \right)}}_2} \cdot {\mathrm{DNAH}}} \right],
\end{eqnarray*}

(14) \begin{eqnarray*}
\frac{{d\left[ {{{{\left( {{\mathrm{Smad}}} \right)}}_2} \cdot {\mathrm{DNAL}}} \right]}}{{dt}} &=& {{\widetilde{k}}_L}{{\left[ {{\mathrm{Smad}}} \right]}^2}\left[ {{\mathrm{DNAL}}} \right] \nonumber\\ &-& {{\widetilde{k}}_{ - L}} \left[ {{{{\left( {{\mathrm{Smad}}} \right)}}_2} \cdot {\mathrm{DNAL}}} \right],
\end{eqnarray*}

In equilibrium, we obtain:


(15) \begin{eqnarray*}
\frac{{\left[ {{\mathrm{Myc}}} \right]\left[ {{\mathrm{DNAH}}} \right]}}{{\left[ {{\mathrm{Myc}} \cdot {\mathrm{DNAH}}} \right]}} = \frac{{{{k}_{ - 1}}}}{{{{k}_1}}} = {{K}_1},
\end{eqnarray*}

(16) \begin{eqnarray*}
\frac{{{{{\left[ {{\mathrm{Myc}} \cdot {\mathrm{DNAH}}} \right]}}^2}}}{{\left[ {{{{\left( {{\mathrm{Myc}} \cdot {\mathrm{DNAH}}} \right)}}_2}} \right]}} = \frac{{{{k}_{ - 2}}}}{{{{k}_2}}} = {{K}_2},
\end{eqnarray*}

(17) \begin{eqnarray*}
\frac{{\left[ {{\mathrm{Myc}}} \right]\left[ {{\mathrm{DNAL}}} \right]}}{{\left[ {{\mathrm{Myc}} \cdot {\mathrm{DNAL}}} \right]}} = \frac{{{{k}_{ - L}}}}{{{{k}_L}}} = {{K}_L},
\end{eqnarray*}

where, ${{K}_1}$, ${{K}_2}$, and ${{K}_L}$ are the equilibrium dissociation constants.

Using Eqns (15) and (16), we obtain:


(18) \begin{eqnarray*}
\frac{{{{{\left[ {{\mathrm{Myc}}} \right]}}^2}{{{\left[ {{\mathrm{DNAH}}} \right]}}^2}}}{{\left[ {{{{\left( {{\mathrm{Myc}} \cdot {\mathrm{DNAH}}} \right)}}_2}} \right]}} = K_1^2{{K}_2} = {{K}_H},
\end{eqnarray*}

In equilibrium, for Smad1, we obtain the following:


(19) \begin{eqnarray*}
\frac{{{{{\left[ {{\mathrm{Smad}}} \right]}}^2}\left[ {{\mathrm{DNAH}}} \right]}}{{\left[ {{{{\left( {{\mathrm{Smad}}} \right)}}_2} \cdot {\mathrm{DNAH}}} \right]}} = \frac{{{{{\widetilde{k}}}_{ - H}}}}{{{{{\widetilde{k}}}_H}}} = {{\widetilde{K}}_H},
\end{eqnarray*}

(20) \begin{eqnarray*}
\frac{{{{{\left[ {{\mathrm{Smad}}} \right]}}^2}\left[ {{\mathrm{DNAL}}} \right]}}{{\left[ {{{{\left( {{\mathrm{Smad}}} \right)}}_2} \cdot {\mathrm{DNAL}}} \right]}} = \frac{{{{{\widetilde{k}}}_{ - L}}}}{{{{{\widetilde{k}}}_L}}} = {{\widetilde{K}}_L}.
\end{eqnarray*}

The conservation of mass equations:


(21) \begin{eqnarray*}
\left[ {{\mathrm{Myc}}} \right] &+& \left[ {{\mathrm{Myc}} \cdot {\mathrm{DNAH}}} \right] + 2\left[ {{{{\left( {{\mathrm{Myc}} \cdot {\mathrm{DNAH}}} \right)}}_2}} \right] \nonumber\\ &+& \left[ {{\mathrm{Myc}} \cdot {\mathrm{DNAL}}} \right] = {{\left[ {{\mathrm{Myc}}} \right]}_{tot}},
\end{eqnarray*}

(22) \begin{eqnarray*}
\left[ {{\mathrm{Smad}}} \right] + 2\left[ {{{{\left( {{\mathrm{Smad}}} \right)}}_2} \cdot {\mathrm{DNAH}}} \right] &+& 2\left[ {{{{\left( {{\mathrm{Smad}}} \right)}}_2} \cdot {\mathrm{DNAL}}} \right]\nonumber\\ &=& {{\left[ {{\mathrm{Smad}}} \right]}_{tot}},
\end{eqnarray*}

(23) \begin{eqnarray*}
\left[ {{\mathrm{DNAH}}} \right] &+& \left[ {{\mathrm{Myc}} \cdot {\mathrm{DNAH}}} \right] + 2\left[ {{{{\left( {{\mathrm{Myc}} \cdot {\mathrm{DNAH}}} \right)}}_2}} \right] \nonumber\\ &+& \left[ {{{{\left( {{\mathrm{Smad}}} \right)}}_2} \cdot {\mathrm{DNAH}}} \right] = {{\left[ {{\mathrm{DNAH}}} \right]}_{tot}},
\end{eqnarray*}

(24) \begin{eqnarray*}
\left[ {{\mathrm{DNAL}}} \right] + \left[ {{\mathrm{Myc}} \cdot {\mathrm{DNAL}}} \right] &+& \left[ {{{{\left( {{\mathrm{Smad}}} \right)}}_2} \cdot {\mathrm{DNAL}}} \right] \nonumber\\\ &=& {{\left[ {{\mathrm{DNAL}}} \right]}_{tot}},
\end{eqnarray*}

where [Myc]*_tot_*, [Smad]*_tot_*, [DNAH]*_tot_*, and [DNAL]*_tot_* are the total concentration of Myc, Smad1, DNAH, and DNAL, respectively.

For the equilibrium dissociation constants of Myc, we adopt the following values [25, 35]: ${{K}_1} = 145{\mathrm{\ nM}}$ and ${{K}_2} = 90{\mathrm{\ nM}}$. We assume here for simplicity that all binding sites within DNAH genomic regions can be characterized by the same effective equilibrium constant. Based on the experimental observation that the vast majority of Myc binding events occur in DNAH regions, Fig. 6B and C, we assume a weaker Myc binding affinity for DNAL regions as compared to DNAH regions (such weaker binding is characterized by a larger equilibrium dissociation constant), ${{K}_L} = 500\ {\mathrm{nM}}$. For Smad1 homodimer binding to DNAH and DNAL we assume, ${{\widetilde{K}}_H} = {{10}^{ - 14}}\ {{{\mathrm{M}}}^2}$ and ${{\widetilde{K}}_L} = 2.5 \cdot {{10}^{ - 14}}\ {{{\mathrm{M}}}^2}$, respectively (the order of magnitude of ${{\widetilde{K}}_H}$ and ${{\widetilde{K}}_L}$ is consistent with the measurements performed in [34]). Qualitatively, the model predictions are robust for a wide range of magnitudes for equilibrium binding constants (Supplementary Figs S9 and S10).

Solving the rate equations at equilibrium, we obtain the dependence of the concentration of bivalent Myc heterotetramer and Smad1 homodimer bound to DNAH, as a function of the total Myc concentration (Fig. 6D and E). Taking into account that the expression level of Myc in ESCs is four times as high as in dEN, while the expression level of Smad1 is maintained constant in both ESCs and dEN (Fig. 6A and Supplementary Table S2 in [23]), the developmental transition from ESCs to dEN corresponds to the shift from right to left along the *x*-axis in Fig. 6D and E. The key observation here is the existence of a sharp transition in the concentration dependence of bound Myc and Smad1, Fig. 6C and D. This sharp transition qualitatively reproduces the shift in the GC-content distribution across binding peaks of c-Myc and Smad1 upon the developmental transition from ESCs to dEN, Fig. 6B and C. Our model thus predicts that this transition is regulated by the concentration of c-Myc, competing with Smad1 for high-GC binding. The sharpness of the predicted transition stems from the cooperative nature of c-Myc-Max bivalent heterotetramer binding to high-GC regions (Supplementary Fig. S9). A conventional model assuming that c-Myc-Max binds DNA only as a heterodimer, Eq. (5), and without assuming Eq. (6), does not predict a sharp transition (Supplementary Fig. S9). Taking together these findings, we predict that first, nonconsensus c-Myc–DNA and Smad1–DNA binding, and second, the formation of a bivalent c-Myc-Max heterotetramer in GC-rich genomic regions, are the two necessary features driving the observed transitions in TF–DNA binding landscape upon developmental transition from ESCs to dEN. It is important to stress that the key prediction of our model is that no direct interaction between c-Myc and Smad1 is required in order to achieve the transition in the TF–DNA binding landscape. This is consistent with previous findings in seminal work by Orkin *et al.*, revealing that c-Myc-centered network is largely independent of the core ESC pluripotency network [12]. Smad1 belongs to this pluripotency network along with Pou5f1 (Oct4), Nanog, Sox2, Srf, and other TFs [12].

We emphasize that, in contrast to crystallographic measurements [25], AlphaFold3 modeling of a bivalent c-Myc-Max heterotetramer was performed using longer, genomic DNA sequences (Fig. 5C and Supplementary Table S3). In this case, c-Myc-Max interaction with DNA leads to DNA looping, resulting in the formation of a bivalent c-Myc-Max heterotetramer (Fig. 5C and Supplementary Table S3). In order to validate the generality of this effect, using AlphaFold3, we tested c-Myc-Max binding to 189 genomic sequences with the length varying from 74-bp to 174-bp (Supplementary Table S3). The sequences were selected from c-Myc MNChIP-seq peaks in ESCs, where each sequence contains two c-Myc specific binding motifs, CACGTG, adjacent to the sequence edges (Supplementary Table S3). We observed that c-Myc-Max forms a bivalent heterotetramer and induces DNA looping for 83% (157 out of 189) of sequences (Fig. 5C and Supplementary Table S3). In addition, AlphaFold3 predicts that the fraction of looped DNA sequences correlates with the DNA length and the GC-content (Supplementary Fig. S8). This suggests that a c-Myc-Max bivalent heterotetramer is abundant in the genome.

## Discussion

First, using a statistical-mechanics model for TF–DNA recognition trained on MNChIP-seq data in ESCs, we revealed that key pluripotency TFs possess bimodal intrinsic DNA recognition specificity characterized by fundamentally different mechanisms in GC-poor and GC-rich genomic regions (Figs 1–3). GC-poor regions are characterized by enhanced *k*-mer specificity, while GC-rich regions show enhanced GC-content specificity (Fig. 2D). We observed that c-Myc binds GC-rich genomic regions strongly depleted of antinucleosomal DNA sequences (Fig. 4). Second, we predicted that c-Myc represents a competitive regulator of TF–DNA binding preferences for key pluripotency factors (Figs 5 and 6). This is consistent with the previous findings revealing that c-Myc-centered network is largely independent of the core ESC pluripotency network containing Smad1, Pou5f1 (Oct4), Nanog, Sox2, Srf, and other TFs [12]. In particular, our thermodynamic model predicts that the direct interaction between c-Myc and Smad1 is not essential for the experimentally observed transition in the TF–DNA binding landscape in the course of ESC differentiation (Figs 5 and 6). Rather, the formation of a bivalent c-Myc-Max heterotetramer (e.g by looping DNA) in GC-rich regions is necessary for achieving this transition (Figs 5C and 6). Past crystallographic studies [25] and our AlphaFold3 modeling provide support for the existence of a bivalent c-Myc-Max heterotetramer (Fig. 5). AlphaFold3 prediction of DNA looping by a bivalent c-Myc-Max heterotetramer represents a significant step toward understanding the molecular mechanism of how c-Myc controls gene expression.

Our results point out that c-Myc might affect enhancer–promoter interactions, especially at sub-kilobase length-scales. Strikingly, it was discovered that despite the fact that repression of cohesin eliminates nearly all loop domains, this does not significantly affect the expression level of the majority (∼90%) of genes in a human cell line [36, 37]. In particular, enhancer–promoter pairs separated by <50 kb are weakly affected by a loss of contact domains and loops [37]. It was also recently revealed that the transcription factor CTCF, and topologically associated domain (TAD) insulation are not required for enhancer–promoter interactions [38]. Rather, it was proposed that TF–DNA interactions might be responsible for establishing enhancer–promoter contacts [38]. Recently described microcompartments were also shown to be largely unaffected by loss of loop extrusion [39]. This suggests that the local, dynamic, three-dimensional conformational ensemble of promoters and enhancer–prompter interactions remains relatively robust despite the elimination of cohesin and CTCF. Our results point out the possibility that transient bivalent (or multivalent) TF–DNA interactions, analogous to a bivalent c-Myc-Max heterotetramer, can stabilize this local, dynamic conformational ensemble of the genome. Consistent with this idea, it was recently identified that p53 drives direct and indirect changes in genome compartments, TADs, and DNA loops [40]. Further experimental studies probing the effect of how c-Myc affects DNA conformations and enhancer–promoter interactions are necessary in order to validate this hypothesis.

## Supplementary Material

gkaf333_Supplemental_Files

## Data Availability

All the data are either provided as supplementary tables or cited.
