# Supplementary material for: Bimodal specificity of TF–DNA recognition in embryonic stem cells
Source: Nucleic Acids Res. 2025 Apr 26;53(8):gkaf333. doi: 10.1093/nar/gkaf333 (PMC12034040; doi:10.1093/nar/gkaf333)
Supplement: gkaf333_Supplemental_Files [file gkaf333_supplemental_files.zip › REVISION_Supp_Information_DL_v10.pdf]

Supplementary Figures

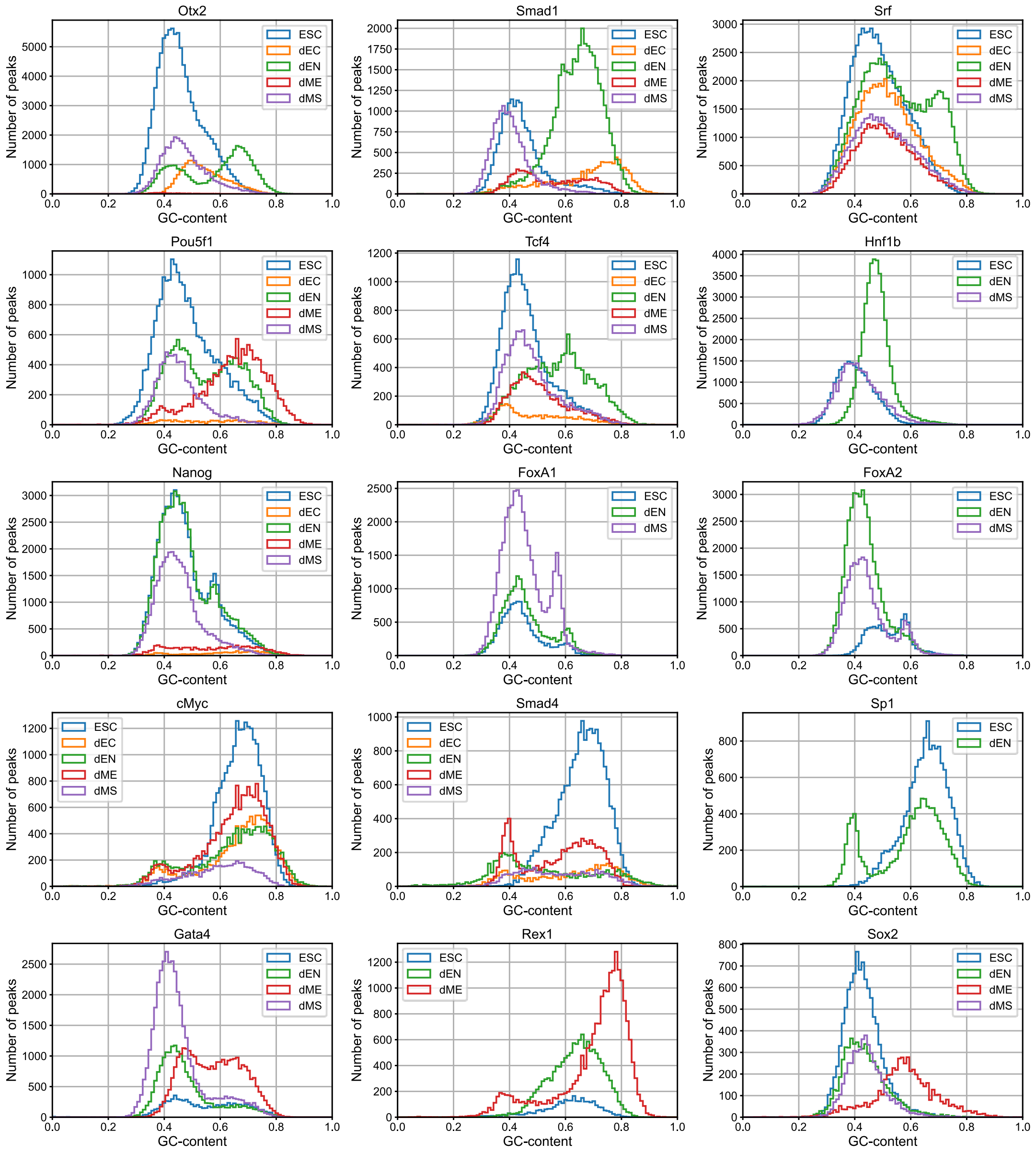

Supplementary Figure S1. GC-content distribution of MNChIP-seq peaks for different TFs in five cell types: ESCs, ectoderm (dEC), endoderm (dEN), mesoderm (dME), and mesendoderm (dMS). Each cell type is marked by a unique color. We binned the GC-content range (0 to 1) into 100 equal segments, tallying the occurrence of binding peaks within these intervals. The selected TFs exhibit a bimodality or/and transition of the GC-content distribution from ESCs to other layers. The first two rows include TFs with the shift of the GC-content distribution from lower to higher values upon the developmental transition from ESCs to dEN: Otx2, Smad1, Srf, Pou5f1, Tcf4, and Hnf1b. The third row contains TFs with a similar bimodal GC-content distribution in ESCs and dEN: Nanog, FoxA1, and FoxA2. The fourth row includes TFs with the shift of the GC-content distribution from higher to lower values upon the developmental transition from ESCs to dEN: c-Myc, Smad4, and Sp1. The last row shows TFs with the shift of the GC-content distribution from lower to higher values upon the developmental transition from ESCs to dME: Gata4, Rex1, Sox2.

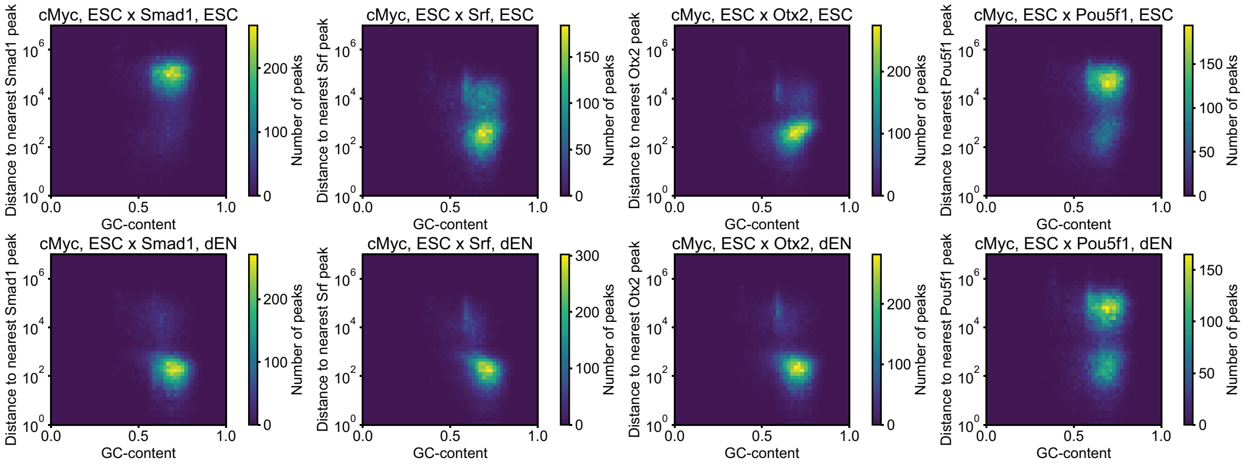

Supplementary Figure S2. The joint distribution of the GC-content in c-Myc binding peaks and their proximity to the nearest peaks of Smad1, Srf, Otx2, and Pou5f1, respectively. The upper row shows the joint distribution between peaks of c-Myc in ESCs and peaks of the other TF also in ESCs, and the bottom row shows the joint distribution between peaks of c-Myc in ESCs and peaks of the other TF in dEN. The bottom row demonstrates that Smad1 in dEN occupies genomic regions that were previously bound by c-Myc in ESCs. This means that Smad1 outcompetes c-Myc upon developmental transition from ESCs to dEN. The distances are measured between peak centers. We partitioned the GC-content range (0 to 1) into 50 equal bins and the distance range (1 to 10^7-bp) into 49 logarithmically scaled bins, counting the frequency of binding peaks within the bins.

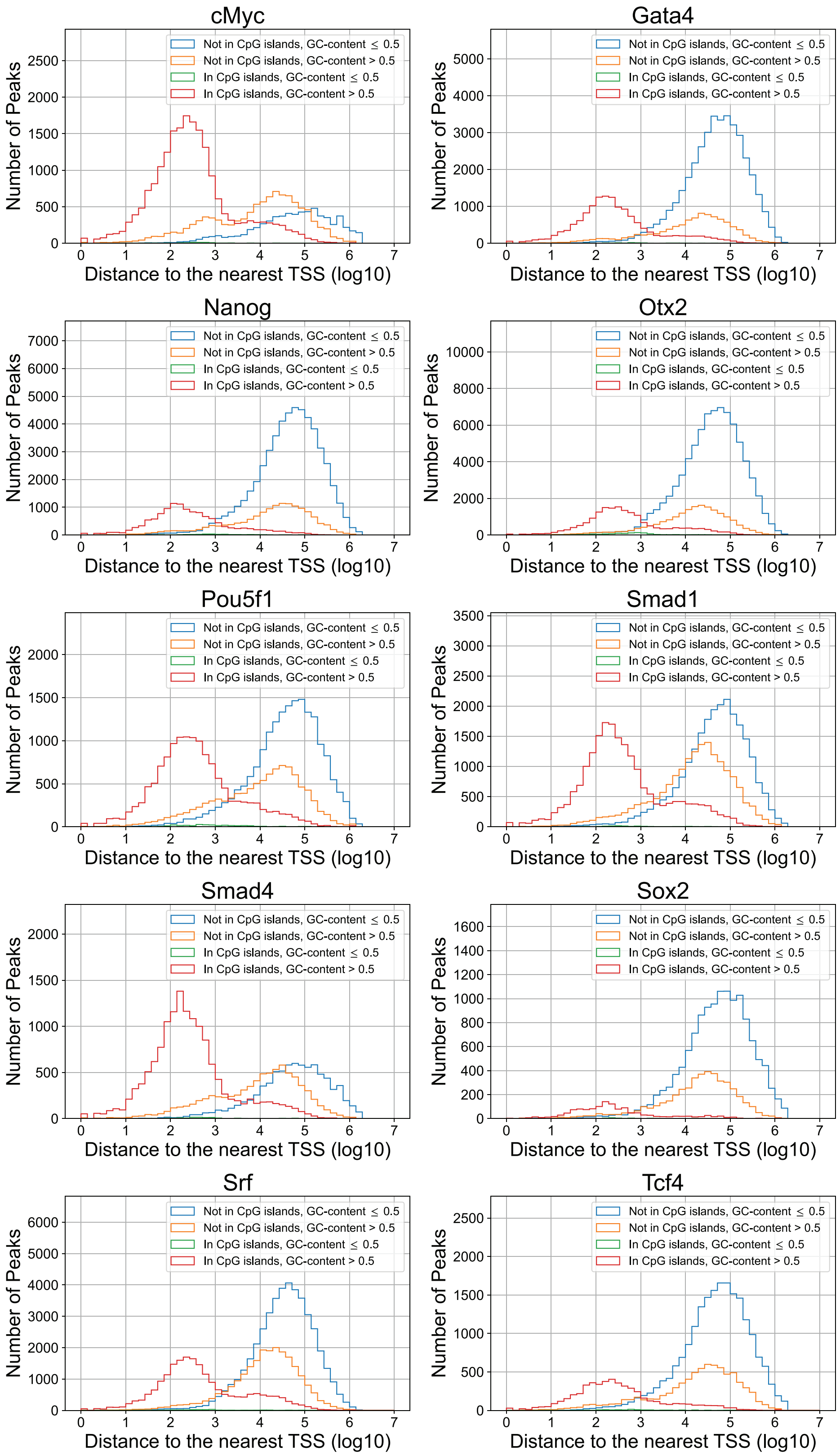

Supplementary Figure S3. Distribution of distances from the MNChIP-seq peak centers to their nearest TSS. MNChIP-seq data is compiled from ref. (2) as described in the main text. The peaks are compiled from five developmental layers and separated into four groups based on their GC-content and association with CpG islands. In case of overlapping peaks from different developmental layers, we included only a peak with the leftmost start coordinate. A peak is considered to be inside a CpG island if it intersects with it by at least 1-bp. The coordinates of CpG islands were downloaded from the Genome Browser in the reference genome hg19 (Supplementary Table S2).

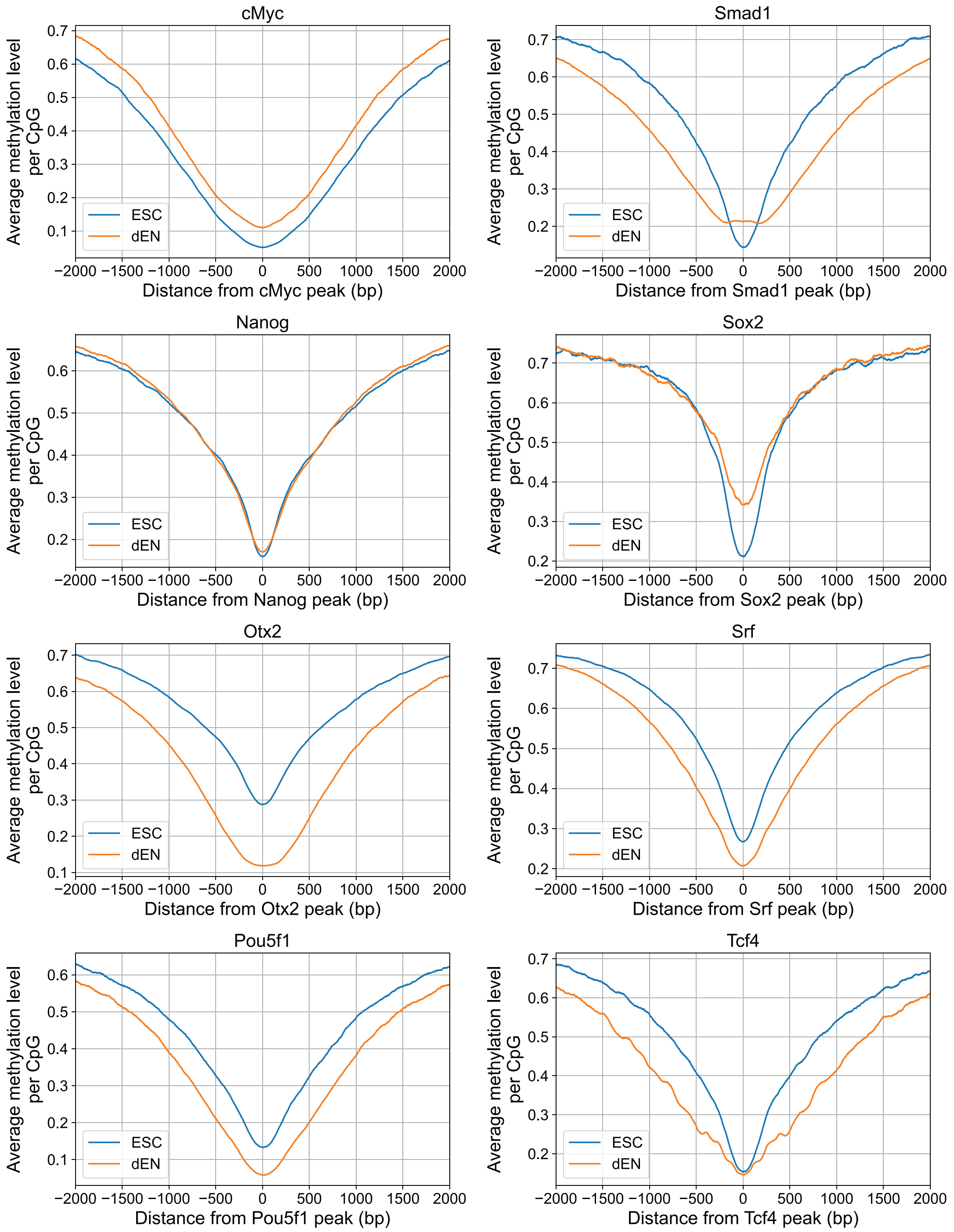

Supplementary Figure S4. Average DNAme level within 4-kb-wide genomic regions aligned by the center of MNChIP-seq binding peaks of the selected TFs in two cell types, ESCs and dEN. Methylation data was taken from refs. (3,4), the accession number GSE46644; we used the replicas GSM1112840_BiSeq_cpgMethylation_BioSam_1122_HUES64 from the GSE46644_bedFiles_set1 archive and GSM916051_BiSeq_cpgMethylation_HUES64_derived_CD184_BioSam_705 from the GSE46644_bedFiles_set2 archive for ESCs and dEN, respectively. The methylation level was normalized by the total number of CpG pairs located at a given position relative to the peak center. The normalized methylation level was then smoothed out using the 100-bp sliding window.

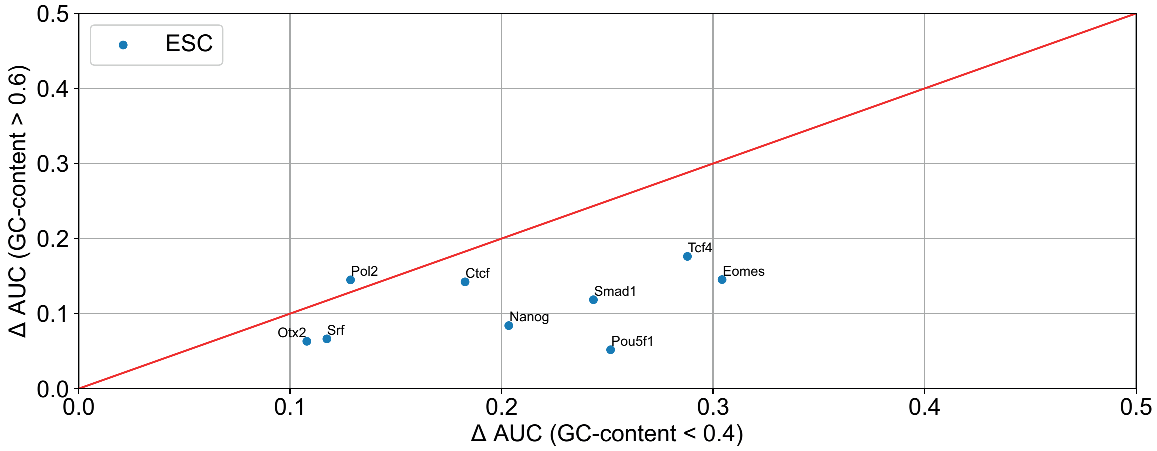

Supplementary Figure S5. Summary of *k*-mer binding specificity depending on the GC-content. For each TF, we compiled two groups of MNChIP-seq peaks from ESCs with GC-content below 0.4 and above 0.6, respectively. In order to compile the control set of sequences (i.e., the 'background'), we used ATAC-seq data from (1), with the GEO accession number GSM3163874. Similar to the peak sequence set, we compiled the ATAC-seq peaks with GC-content below 0.4 and above 0.6, respectively. We discarded ATAC-seq peaks which overlapped with MNChIP-seq peaks. We calculated the area under the ROC curve (AUC) in both groups for different *k*-mer length *k*, and then computed *k*-mer specificity characteristic, ΔAUC, as described in the main text. The pairs of ΔAUC values for different GC-content were used as coordinates to build a scatter plot. Each point is labeled by the corresponding TF. If the number of peaks in either of the two groups is less than 1000, the point is not shown. The overwhelming majority of proteins, 8 out of 9, fall below the diagonal.

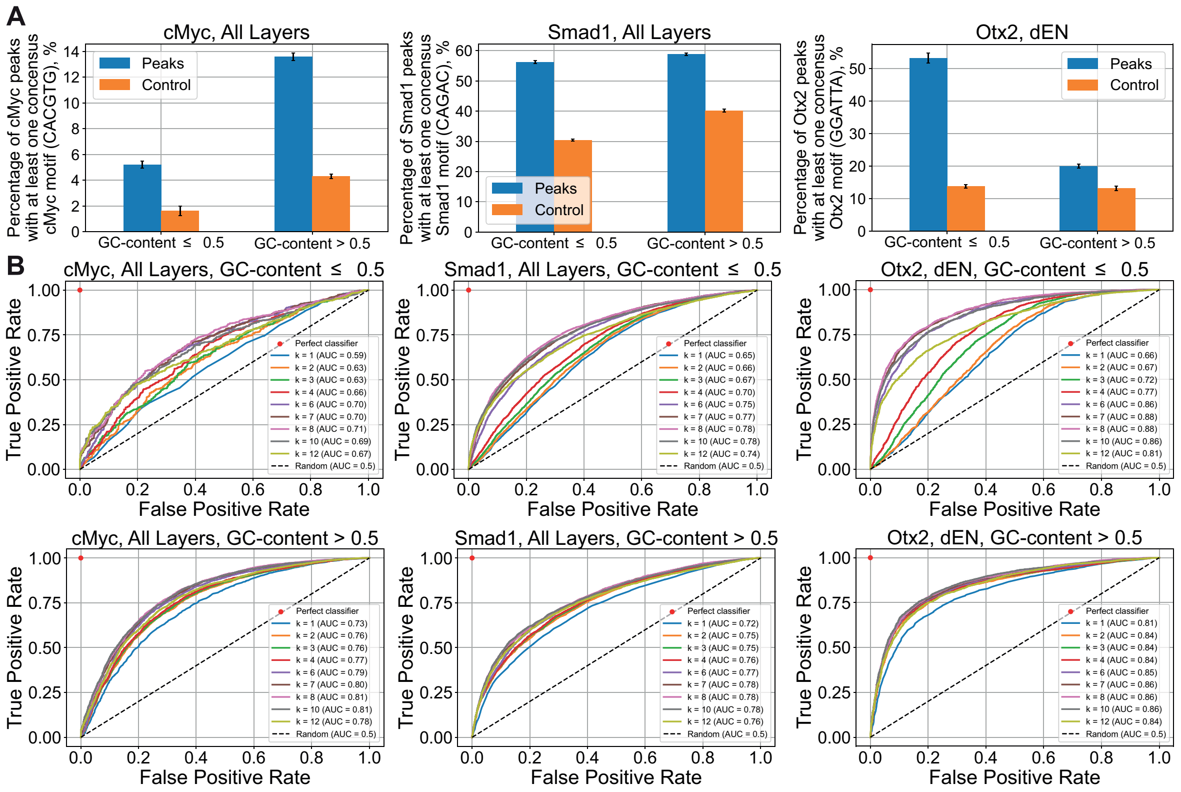

Supplementary Figure S6. (**A**) Example: Fraction of MNChIP-seq peaks containing specific, consensus TF binding motifs in GC-rich and GC-poor genomic regions. Fraction of MNChIP-seq peaks of c-Myc, Smad1, and Otx2 (blue) containing at least one specific, consensus motif, CACGTG, CAGAC, and GGATTA, respectively, compared with the corresponding set of control sequences (orange). For c-Myc

and Smad1, we compiled MNChIP-seq peaks from all five developmental stages (ESCs, dMS, dME, dEN, and dEC), while for Otx2 from dEN only. In case when peaks from different stages overlap, we included only a peak with the leftmost start coordinate. We divided the peaks into two groups, GC-rich (GC-content > 0.5) and GC-poor (GC-content ≤ 0.5). Motif counting was performed along the entire width of each peak. For control, we took non-overlapping genomic sequences located 100-bp upstream and 100-bp downstream from each peak, respectively. We term this control as the 'nearest-neighbor' control. The length of each control sequence is identical to the width of the nearest peak. For each sequence group (i.e., GC-rich and GC-poor), we retain only control sequences that match the GC-content threshold of the group. The resulting dataset consists of 6631 c-Myc peaks with GC-content ≤ 0.5, and 30506 peaks with GC-content > 0.5; the control dataset consists of 9108 sequences with GC-content ≤ 0.5, and 23019 sequences with GC-content > 0.5. For Smad1, the resulting dataset consists of 23939 peaks with GC-content ≤ 0.5 and 37812 peaks with GC-content > 0.5; the control dataset consists of 39671 sequences with GC-content ≤ 0.5 and 27780 sequences with GC-content > 0.5. For Otx2 in dEN, the resulting dataset consisted of 11747 peaks with GC-content ≤ 0.5 and 23997 peaks with GC-content > 0.5; the control dataset consists of 20652 sequences with GC-content ≤ to 0.5 and 17693 sequences with GC-content > 0.5. To calculate error bars for each sequence group, we randomly split the group into five subgroups, computing the fraction of peaks containing at least one consensus motif for each subgroup. Finally, we calculate the mean and standard deviation of the fraction of peaks containing the motif across subgroups. Error bars represent one standard deviation in each direction around the mean. (**B**) Receiver Operating Characteristic (ROC) curves visualizing the ability of the proposed energy model to predict c-Myc, Smad1, and Otx2 binding peaks depending on the *k*-mer length and GC-content. For computing ROC curves, we used the same sets of genomic and control sequences as in (**A**). We preprocessed these sequences by taking the central 100-bp from each sequence and removing the sequences with unidentified bases (labeled by N). The prediction of whether the sequence belongs to the peak or the background is based on the value of its free energy defined in the main text. Precomputations of binding energies were made using 80% of the MNChIP-seq dataset, and the remaining 20% of the dataset was used to evaluate the model performance. The *x*-axis shows a false positive rate (FPR), and the *y*-axis shows a true positive rate (TPR). Pairs of TPR and FPR at different threshold values of the free energy constitute a ROC curve for each *k*-mer length. The diagonal dotted line represents random classifiers, assigning peak status with the same probability for peak and control sequences. Perfect classifier is represented by a red dot at the coordinates (0,1). The area under the curve (AUC) provides an estimate for the probability that a randomly selected peak sequence has a lower free energy than a randomly selected background sequence. Higher values of AUC indicate superior predictive capability.

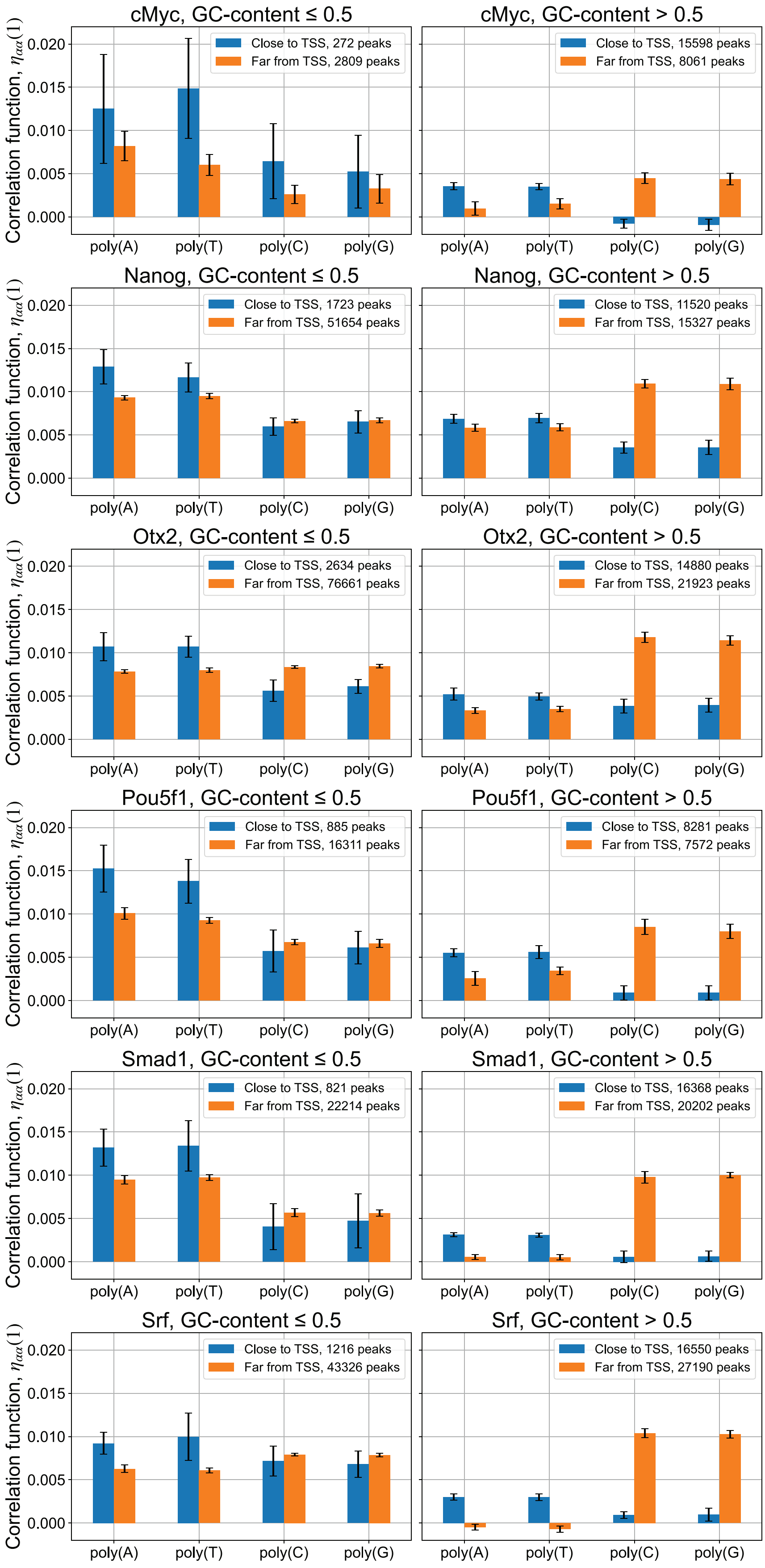

Supplementary Figure S7. Enrichment of poly(A), poly(T), poly(C), and poly(G) in DNA sequences inside MNChIP-seq peaks for c-Myc, Nanog, Otx2, Pou5f1, Smad1, and Srf. Computed $\eta_{AA}(1)$, $\eta_{TT}(1)$, $\eta_{CC}(1)$, and $\eta_{GG}(1)$ for MNChIP-seq peaks of each TF, compiled from all five developmental stages of

ESCs. Correlation functions were computed for 100-bp-long DNA sequences extracted from the center of peaks. In case of overlapping peaks from different stages, we included only a peak with the leftmost start coordinate. Peaks shorter than 100-bp were omitted from the analysis. Left and right plots represent correlation functions computed for GC-poor (GC-content<0.5) and GC-rich (GC-content>0.5) MNChIP-seq peaks, respectively. Peaks located within the interval +/-1000-bp are termed 'close to TSS'. Peaks located outside of the interval +/-1000-bp around TSS are termed 'far from TSS'. To compute error bars for each sequence group, we randomly split the group into ten subgroups with an equal number of sequences in each subgroup and computed the mean correlation functions, $\eta_{\alpha\alpha}(1)$, for each subgroup. Error bars represent one standard deviation in each direction around the mean.

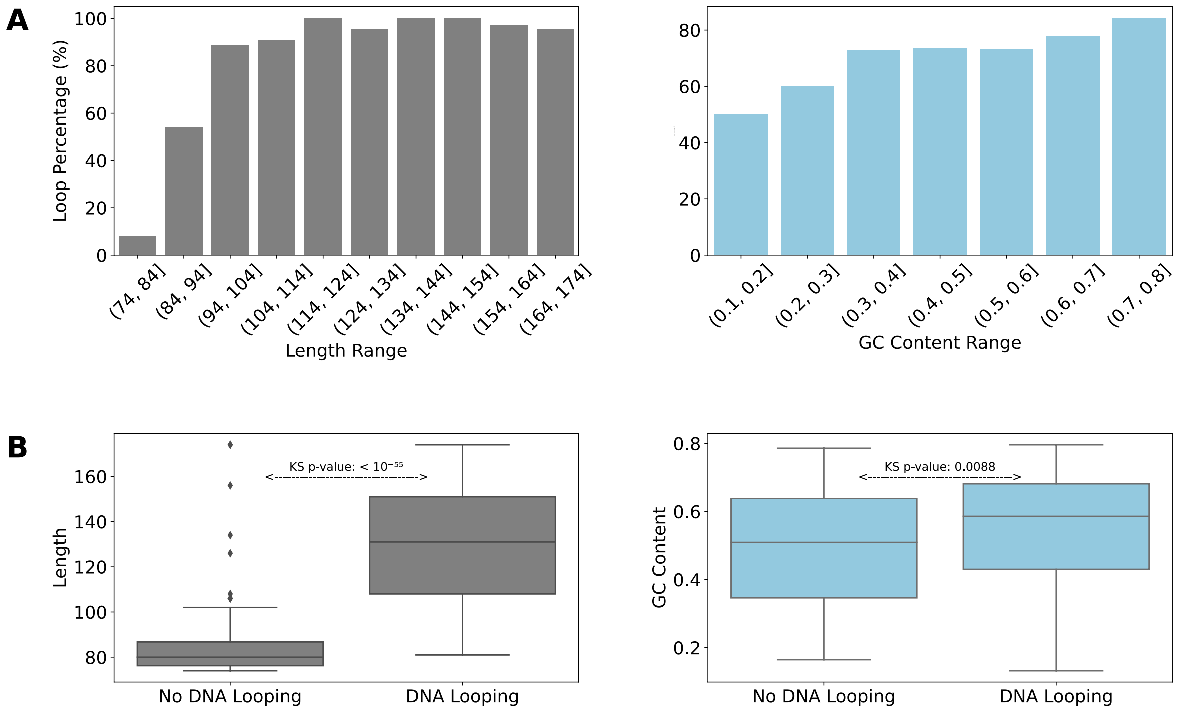

Supplementary Figure S8. AlphaFold3 predicts the formation of a bivalent c-Myc-Max heterotetramer through DNA looping as a function of DNA sequence length and GC-content. (**A**) Left: Histogram for the fraction of genomic DNA sequences forming a bivalent c-Myc-Max heterotetramer by DNA looping binned as a function of the sequence length. Overall, 463 genomic DNA sequences within the length range 74—174-bp were simulated. Among those 463 sequences, 181 are extracted from c-Myc MNChIP-seq peaks in ESCs, 62 from c-Myc peaks in Mesendoderm (dMS), and 220 from outside MNChIP-seq peaks. Each DNA sequence contains two specific c-Myc binding sites (CACGTG) located at the opposite ends of the sequence: AATCCGCACGTGGCCTGGTTCGCCTGGAAGCTGCCA AAGCGAAGGCAAGAGAGCCCGGGACCCTGCTGTAAAACAGCAAGCAGCACGTGTCCCAG. Each sequence contains 6-bp-long overhangs outside of the specific motif at the sequence ends. The higher the DNA length is, the higher the fraction of looped DNA molecules. Right: the same data as in the Left plot, but binned as a function of the GC-content. The higher the GC-content is, the higher the fraction of looped DNA molecules. (**B**) The same data as in (**A**), represented as a box plot. The computed two-sample Kolmogorov-Smirnov p-value demonstrates that both the DNA length and the GC-content can statistically significantly distinguish between looped and non-looped DNA molecules. However, the DNA length represents a much stronger predictor compared to the GC-content.

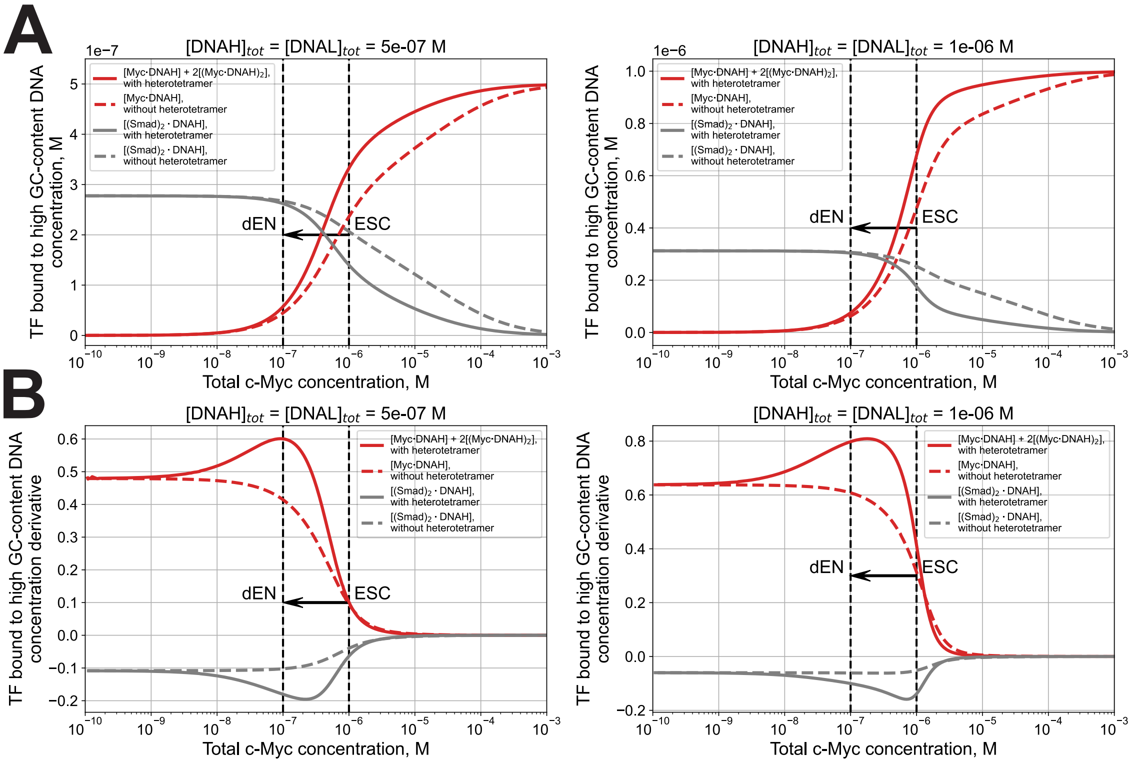

Supplementary Figure S9. Comparison of the model assuming the formation of a bivalent c-Myc-Max heterotetramer in high-GC-content DNA regions with the conventional model without such an assumption. Only the model assuming the formation of a bivalent heterotetramer explains the sharp transition of the Smad1 binding intensity from low- to high-GC-content regions upon the developmental transition from undifferentiated ESCs to endoderm (dEN). (**A**) The concentration of the high-GC-content DNA (DNAH) bound by c-Myc-Max (both as a heterodimer and as a bivalent heterotetramer) and by Smad1 homodimer computed using the model assuming the formation of a bivalent c-Myc-Max heterotetramer in high-GC-content DNA regions (solid lines), and using the conventional model without such an assumption (dashed lines). The concentration is shown as a function of the total c-Myc-Max concentration (i.e., the sum of the concentration of DNAH bound by c-Myc-Max heterodimer and a bivalent c-Myc-Max heterotetramer), using two values of the total DNAH and DNAL concentrations, $[\mathrm{DNAH}]_{tot}$=$[\mathrm{DNAL}]_{tot}$=0.5 μM (left), and $[\mathrm{DNAH}]_{tot}$=$[\mathrm{DNAL}]_{tot}$=1 μM (right). The following values were used for the remaining model parameters: $[\mathrm{Smad}]_{tot} = 1\mu\mathrm{M}$, $K_1 = 145\mathrm{nM}$, $K_2 = 90\mathrm{nM}$, $K_L = 500\mathrm{nM}$, $\widetilde{K}_H = 10^{-14}\ \mathrm{M}^2$, $\widetilde{K}_L = 2.5 \cdot 10^{-14}\ \mathrm{M}^2$. Dashed vertical lines qualitatively represent c-Myc concentrations in ESCs (right) and dEN (left), respectively. The arrow illustrates the developmental transition from ESCs (with a higher c-Myc concentration) to dEN (with a lower c-Myc concentration). (**B**) The computed derivatives of the concentration functions shown in (**A**) demonstrate that only the model assuming the formation of a bivalent c-Myc-Max heterotetramer predicts a sharp transition of the Smad1 binding intensity upon the developmental transition from ESCs to dEN.

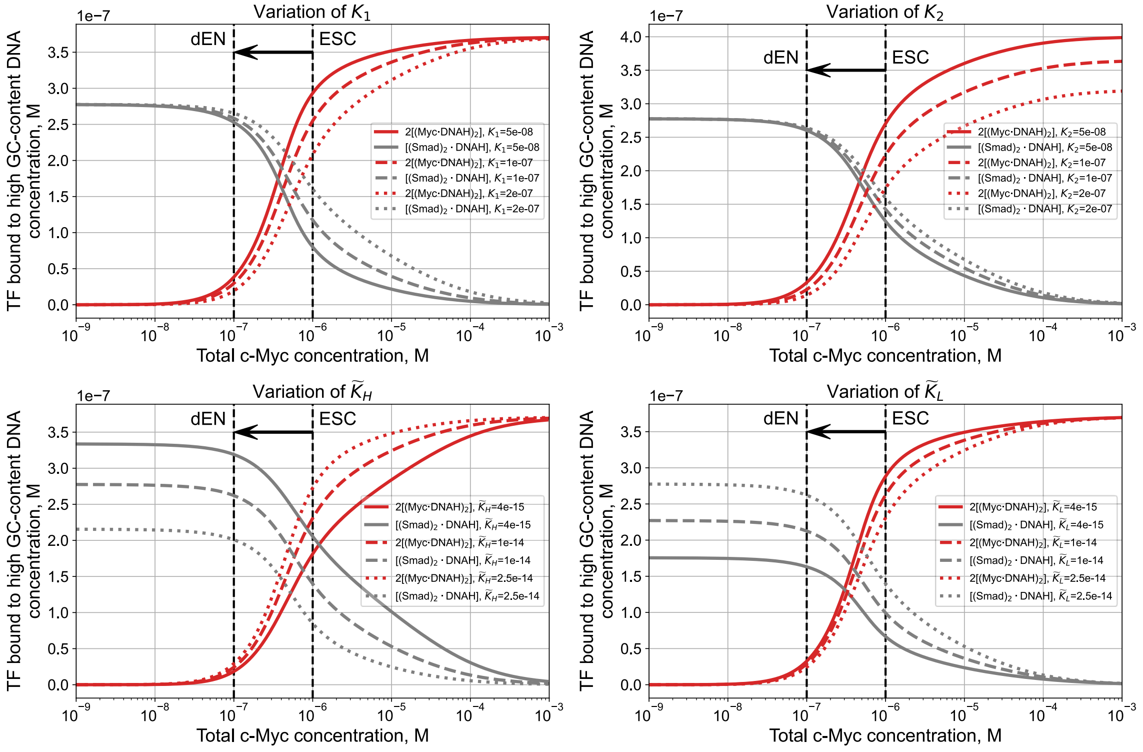

Supplementary Figure S10. Robustness of the proposed kinetic model with respect to parameter variation. The computed concentration of the high-GC-content DNA (DNAH) bound by a bivalent c-Myc-Max heterotetramer and by Smad1 homodimer are shown as a function of the total c-Myc concentration, for different values of the parameters indicated above each plot. Each graph represents the variation of one parameter, namely K_1, K_2, $\widetilde{K}_H$, and $\widetilde{K}_L$. For the fixed parameters in each case, we used the following values: $K_1 = 145\text{nM}$, $K_2 = 90\text{nM}$, $\widetilde{K}_H = 10^{-14}\ \text{M}^2$, and $\widetilde{K}_L = 2.5 \cdot 10^{-14}\ \text{M}^2$. The following values were used for the remaining model parameters: $[\text{DNAH}]_{tot} = [\text{DNAL}]_{tot} = 0.5\ \mu\text{M}$, $[\text{Smad}]_{tot} = 1\mu\text{M}$, $K_L = 0.5\ \mu\text{M}$.

Supplementary Tables (External Excel Files)

Supplementary Table S1. Summary of MNChIP-seq data from ref. (2) used in the analysis. The table specifies which replica was used for each TF in each cell type, and the number of peaks it contained.

Supplementary Table S2. Coordinates of CpG islands in the reference genome hg19. The data was downloaded from the eponymous track at the Genome Browser (https://genome.ucsc.edu/cgi-bin/hgTables?db=hg19&hgta_group=regulation&hgta_track=cpgIslandExt&hgta_table=cpgIslandExt&hgta_doSchema=describe+table+schema). We used the following columns: (1) chrom – reference sequence chromosome or scaffold, (2) chromStart – start position of CpG island in chromosome, and (3) chromEnd – end position of CpG island in chromosome.

Supplementary Table S3. (**Sheet 1**) Summary of 189 genomic sequences extracted from c-Myc MNChIP-seq peaks in ESCs, used in AlphaFold3 simulations. The length of the sequences is varying within the interval from 74-bp to 174-bp. Each sequence contains two c-Myc specific binding motifs, CACGTG, adjacent to the sequence edges. The motifs are located 6-bp away from the sequence edges. We observed that c-Myc-Max forms a bivalent heterotetramer inducing DNA looping for 83% (157 out of 189) of sequences. (**Sheet 2**) Sequences used for a control. We randomly reshuffled two sequences (with peak ID 820 and peak ID 1060), producing for each sequence 10 randomized replicas containing the specific binding motif intact, and another 10 randomized replicas reshuffling the entire sequence,

including the specific motif. For sequence ID 820, 100% (10 out of 10) randomized replicas containing the intact motif, and 10% (1 out of 10) containing the reshuffled motif formed DNA loops, respectively. For sequence ID 1060, 80% (8 out of 10) randomized replicas containing the intact motif, and 0% (0 out of 10) containing the reshuffled motif formed DNA loops, respectively. (**Sheet 3**) Protein sequences we used in all simulations.

1. Inoue, F., Kreimer, A., Ashuach, T., Ahituv, N. and Yosef, N. (2019) Identification and Massively Parallel Characterization of Regulatory Elements Driving Neural Induction. *Cell Stem Cell*, **25**, 713-727.e710.
2. Tsankov, A.M., Gu, H., Akopian, V., Ziller, M.J., Donaghey, J., Amit, I., Gnirke, A. and Meissner, A. (2015) Transcription factor binding dynamics during human ES cell differentiation. *Nature*, **518**, 344-349.
3. Ziller, M.J., Gu, H., Müller, F., Donaghey, J., Tsai, L.T.Y., Kohlbacher, O., De Jager, P.L., Rosen, E.D., Bennett, D.A., Bernstein, B.E. *et al.* (2013) Charting a dynamic DNA methylation landscape of the human genome. *Nature*, **500**, 477-481.
4. Gifford, C.A., Ziller, M.J., Gu, H., Trapnell, C., Donaghey, J., Tsankov, A., Shalek, A.K., Kelley, D.R., Shishkin, A.A., Issner, R. *et al.* (2013) Transcriptional and epigenetic dynamics during specification of human embryonic stem cells. *Cell*, **153**, 1149-1163.
